# Bikeability cycle training: a route to increasing young people’s subjective wellbeing? A retrospective cohort study

**DOI:** 10.1186/s12889-025-23838-2

**Published:** 2025-08-18

**Authors:** Daniel T. Bishop, Aina Digaeva

**Affiliations:** 1 Centre for Cognitive and Clinical Neuroscience, Department of Life Sciences, Brunel University London, Kingston Lane, Uxbridge, Middlesex UB8 3PH UK; 2 The Bikeability Trust, PO Box 1494, Cambridge, CB22 3YT UK

**Keywords:** Active travel, Adolescent, Children, Cycling, Health, Life satisfaction, Physical activity

## Abstract

**Introduction:**

Increasing the population’s subjective wellbeing is an explicit aim of current UK government policies. The wellbeing of children and young people in the UK is deteriorating, and less than half of them meet national physical activity guidelines, despite the demonstrable benefits of physical activity for wellbeing. Hence, it is important to identify economically viable and effective public health interventions to increase young people’s physical activity, and consequently, their wellbeing. Bikeability cycle training may be such an intervention.

**Methods:**

205 young people aged 11–18 years in UK secondary schools completed an online survey about their subjective wellbeing, their active travel behaviour, and their physical activity levels. They also indicated whether they had undertaken Bikeability Level 2 cycle training when they were between 9 and 11 years of age; retrospective groups were formed on this basis. Their parents/carers (hereafter, ‘parents’) reported their own cycle training status, their active travel behaviour, and their satisfaction with their living circumstances, both at the time of the survey and when their child was 10 years old (Bikeability Level 2 cycle training is delivered to 9-11-year-olds). After screening, complete datasets from 201 young person-parent dyads were retained for analysis.

**Findings:**

Continuous data were analysed via t tests, ANOVAs and nonparametric equivalents; categorical data were analysed using chi-square tests. One hundred-and-thirteen young people who had completed Bikeability Level 2 cycle training reported greater subjective wellbeing than the 88 individuals who had not, on two established measures of wellbeing. They were also more likely to make journeys by cycling and walking, although there were no between-group differences in self-reported moderate-to-vigorous physical activity, sedentariness, nor attitudes towards cycling. Young people who cycled at least once a week reported greater wellbeing than those who never cycled or who only did so once or so a year. Parents who had completed cycle training cycled more frequently than their untrained counterparts, although no differences in walking frequency emerged. Young person and parent attitudes towards cycling were correlated, as were parents’ satisfaction with their current living circumstances and the young people’s subjective wellbeing.

**Conclusions:**

The present data suggest that Bikeability Level 2 graduates are more likely to report greater subjective wellbeing, and to travel by cycling or walking, than those who did not complete Bikeability training. Given the multiple benefits that active travel may confer to a wellbeing economy, these findings warrant further investigation.

**Supplementary Information:**

The online version contains supplementary material available at 10.1186/s12889-025-23838-2.

## Introduction

The UK is witnessing a decline in children and young people’s wellbeing: national statistics show that almost a third of 16–24-year-olds reported some evidence of anxiety or depression between 2017 and 2018 [64], and approximately 17% of children aged 5 to 16 years experienced a mental health problem in 2020 – more than a 50% rise from 2017 [60]; long-term mental health conditions in children and young people have increased over the same period [67]. The latest Good Childhood Report [84], which draws on data from multiple national data sources, shows that UK 15-year-olds’ life satisfaction deteriorated from 2015 to 2022 and is now lower than that of 27 other European countries.

Socioeconomic inequality may be a reason for this deterioration. Recent evidence suggests a relationship between income and mental health not only for adults [69], but also for adolescents: young people whose household incomes are in the lowest quintile are more likely to be depressed than those in the upper quintile [66]. However, data obtained from 32,676 Year 6 (10-11-year-olds) and Year 9 (13-14-year-olds) pupils in UK schools suggest that, whilst there was a noticeable decline in children and young people’s self-reported emotional wellbeing from 2019 to 2022, this was not correlated with level of deprivation, as measured by the Index of Multiple Deprivation [37]. That said, mental health disorders are also more prevalent in young people who are carers [25], a role that is more common in low-income households [89]. Relatedly, the transition to secondary school can also be detrimental to young people’s wellbeing, if they are moving from a relatively low socioeconomic status primary school to a comparative high status secondary school [58]. Other aspects of young people’s living conditions that influence their wellbeing include fuel poverty [68], lack of security in their home [40], and reductions in physical activity post-Covid [35].

Subjective wellbeing, a term introduced by [28] may be defined as “a person’s cognitive and affective evaluations of his or her life as a whole” [29]. The World Health Organisation [91] describes wellbeing as “…a positive state experienced by individuals and societies. Similar to health, it is a resource for daily life and is determined by social, economic and environmental conditions.” The notion that wellbeing is ultimately experienced by a society, culminating from the individual wellbeing of its constituent members, has led to increasing political interest in measuring subjective wellbeing, to the extent that it now informs government policy. In 2012, Dolan and Metcalfe [31] proposed how data obtained via self-report measures of subjective wellbeing might inform government policy, highlighting three broad approaches to measuring the construct: *evaluative*, global assessments of one’s life such as life satisfaction; *experiential*, an individual’s momentary feelings; and *eudemonic*, the extent to which our basic psychological needs of autonomy (our need to feel we have choices and make independent decisions), competence (our need to feel a sense of mastery and accomplishment) and relatedness (our need to feel a sense of belonging and connection to others) are fulfilled. Consequently, the UK Office for National Statistics [63] employs four questions recommended by Dolan and Metcalfe for monitoring trends in personal wellbeing: (1) *Overall*,* how satisfied are you with your life nowadays?*, (2) *Overall*,* how happy did you feel yesterday?*, (3) *Overall*,* how anxious did you feel yesterday?*, and (4) *Overall*,* how worthwhile are the things that you do in your life?* These four items are now integral to surveys administered by over twenty governmental departments and organisations in the UK, including The Department of Health, The Cabinet Office, and the Higher Education Policy Institute.

Moreover, the evaluative approach described above forms the basis of the *well-being-adjusted life-year*, or WELLBY, which has an estimated monetary value: a one-point change in response to the life satisfaction item above, via a response scale ranging from zero (‘Not at all’) to 10 (Completely), equates to approximately £10,000–16,000, with a midpoint of £13,000 [24]. Using this information, the effects of governmental policies that could enhance well-being can be monetized. Although a *wellbeing economy *– one that is viewed as serving, not benefiting from, social, health, cultural, equality and ecological outcomes – is a more utopian ambition [55], the WELLBY is now a feature of His Majesty’s Treasury’s Green Book [38] as a measure of benefit in social welfare analyses and hence supports governmental policy decision-making [32].

Evidence for the efficacy of physical activity as a mental wellbeing intervention is abundant [9, 19, 33, 39, 49]. Accordingly, data from Sport England’s latest Active Lives Children and Young People survey [79], which also reflects multiple national data sources, show that young people aged 11–16 years completing 30 min or more of physical activity per day were happier, more satisfied with their lives, and felt that things they do in their lives were more worthwhile, than their less active peers – although the relationship between physical activity and wellbeing may be bidirectional [44]. Similarly, using ecological momentary analysis, Bourke and colleagues [17] recorded 119 adolescents’ *core affect* (valence, energetic arousal, tense arousal; [71]) and their life satisfaction (*“all things considered*,* how satisfied are you with your life as a whole today?”*) multiple times per day over a four-day period, along with accelerometer-recorded and self-reported physical activity data. They found that participants’ life satisfaction was positively related to their physical activity levels, whether device-measured or self-reported, and this was mediated by their momentary affective states, i.e., their experiential wellbeing.

Despite such evidence, less than 50% of children and young people in the UK are classified as physically active [79]. The barriers to physical activity are manifold, but they include a perceived lack of social support [75, 76], unrealistic expectations regarding the consequences of increased activity [34] a progressive decline in positive attitudes towards sport and physical activity as children approach adolescence [79], and minimal opportunities for physical activity in the built environment [8, 82] – which may, or may not, be linked to deprivation [6, 43, 52, 53]. Relatedly, one of the most frequently cited barriers to regular exercise – physical activity that is *planned*,* structured and repetitive* [22] – is a perceived lack of time to do so [5, 48, 70]. However, mental health interventions that can be integrated into young people’s daily routines are seemingly the most effective [85]. Because active travel is the most common means of transport for getting to school in the UK [79], it presents an opportunity to incorporate physical activity into young people’s everyday lives, potentially enhancing their wellbeing, at negligible cost.

Higher levels of active travel are associated with increased moderate-to-vigorous physical activity [18, 45, 73], and recent research suggests that children’s and parents’ attitudes towards active travel are generally positive [42], but evidence for the effects of school travel on children’s physical activity levels is inconclusive [78]. For example, Stark and colleagues [80] surveyed 152 Austrian primary schoolchildren, and interviewed 31 of their parents, to ascertain their transport-related attitudes, their travel behaviour and their psychological wellbeing. The authors assessed the children’s psychological wellbeing using the following item: “How do you feel in the first school lesson, when you [have walked; went by bicycle/scooter/bus/train; or were taken by car] to school?”. Children responded to the same item a second time, but in relation to the last, not first, lesson of the day. The researchers found that children’s attitudes towards active travel were more positive than they were for being driven to school or using public transport. However, the children’s psychological wellbeing correlated positively with the frequency with which they used any mode of travel, active or passive. That said, correlations were strongest for walking and scooting, although there was no correlation between cycling frequency and psychological wellbeing. Importantly, parents’ perceptions of their child’s wellbeing were more positive for days on which their child had travelled actively (i.e., they had walked, cycled, or scooted).

Lately, there have been calls for active travel to become a key part of UK public health interventions, from academe [47] and government [87]. However, interventions focused on transport infrastructure changes have only been minorly successful [1, 3]. For example, Aldred and colleagues [3] reported three years’ worth of data that were collected after implementation of three ‘Mini-Holland’ schemes established within three London boroughs – schemes in which walking and cycling-supportive infrastructure was provided, in a bid to increase active travel behaviour in their respective communities. Although these changes led to significant increases in walking, cycling did not increase. Nonetheless, the authors estimated that the economic benefit over a 20-year period would be approximately £9 million for each £1 million invested – a ninefold return on investment. However, Aldred and colleagues [2] identified that long-term barriers to cycling investment include paucities of funding and leadership. Therefore, an educational approach may be a cost-effective complement to infrastructural changes, to increase young people’s active travel behaviour and consequently improve their wellbeing. Recent evidence suggests that school-based interventions designed to increase physical activity may be ineffective [54], and school-based mental health interventions similarly so [20] – but interventions focused on cycling may have potential, not least for increasing motivation to cycle [23].

*Bikeability* (https://www.bikeability.org.uk), the UK government-funded cycle training programme, is a behaviour change intervention that is delivered to over four-hundred thousand children every year, at a cost of £55 per child. The aim of Bikeability training is to give everyone the confidence to cycle and enjoy this skill for life. Accordingly, Bikeability Level 2 training is delivered to 9-11-year-olds on roads and focuses on the four key skills of the UK National Standard for Cycle Training: making good and frequent observations, communicating intentions clearly to other road users, choosing and maintaining the most suitable riding positions, and prioritising road use, particularly at junctions. However, evidence for the efficacy of Bikeability training is limited [41], although a recent independent report by TRL [86] suggests that, as the rate of Bikeability Level 2 training in English local authorities increases, the number of people killed or seriously injured (KSIs) on roads decreases. In other words, *higher* levels of Bikeability delivery were associated with *fewer* KSIs.

In addition, ongoing Active Travel England-funded research shows that young people who completed Bikeability Level 2 cycle training in their primary schools are more competent cyclists, are more likely to cycle for fun or to get somewhere, have more positive attitudes towards cycling on roads, and have a better understanding of the 4 Key Skills of the National Standard for Cycle Training [26] – up to four years after completing the training (Bishop et al., unpublished observations). For these reasons, Bikeability Level 2 cycle training for children may be a suitable intervention to increase their use of cycling for active travel as they move into adolescence.

### The present study: aims, objectives and hypotheses

To our knowledge, there have been no investigations of the long-term effects of cycle training for children on their active travel behaviour and subjective wellbeing when they are young people. The aim of the present study was to determine whether young people who completed Bikeability Level 2 cycle training in their primary schools (‘graduates’) would report higher levels of subjective wellbeing, compared to those who did not complete Level 2 training. We also sought to examine differences between these two groups of young people, in their use of cycling and walking to make journeys, and their levels of moderate-to-vigorous physical activity (MVPA) and sedentariness, because of the apparent relationship between activity levels and active travel behaviour [45]. Considering the influence of living conditions on young people’s subjective wellbeing [40], we also included a measure of parents’ satisfaction with their living circumstances at the time of the survey, and when their child was eligible to undertake Bikeability Level 2 cycle training (aged 9–11 years). Additionally, we sought to reconcile children’s and parents’ attitudes and behaviour vis-à-vis cycling, given recent evidence for the influence of parental cycling attitudes and behaviour on their child’s cycling behaviour [11].

We hypothesised that (1) Bikeability Level 2 graduates would report greater subjective wellbeing than those who did not complete the training; (2) graduates would also report higher levels of active travel (cycling and walking), higher levels of MVPA and lower levels of sedentariness; (3) there would be a positive association between parental cycling attitudes and behaviour and those of their children; (4) parents’ satisfaction with their current living circumstances would correlate positively with the children’s subjective wellbeing; and (5) there would be no differences in satisfaction with living circumstances between the two groups.

## Methods

### Participants

Two-hundred-and-five young people (mean age = 12.9 yrs, SD = 1.6 yrs; median age = 13 yrs) and their parents/carers (hereafter, ‘parents’; mean age = 43.9 yrs, SD = 6.8 yrs; median age = 43 yrs), based in schools and local communities across England, completed the survey, in locations of their choosing. One-hundred-and-eight of the children were female, 93 were male, and one was non-binary; three preferred not to say. One-hundred-and-fifty-nine of the parents were female, 43 were male; three preferred not to say.

One-hundred-and-fifteen of the children had completed their Bikeability Level 2 cycle training, 90 participants had not; they were subsequently grouped on this basis. All 115 children who had completed Bikeability Level 2 cycle training reported that they could cycle, whereas 75 (83.3%) of those who had not completed Bikeability Level 2 cycle training could cycle – a statistically significant association, χ^2^(1, *N* = 205) = 20.68, *p* <.001, Phi = 0.32. Eighty-three parents had completed formal cycle training as a child, 12 had completed it as an adult; 110 had not received any cycle training. Table 1 illustrates the characteristics of the two groups, including physical and mental impairments, for both children and parents.

**Table 1:** Participant Characteristics, by group

		Child		Parent	
		Bikeability Level 2 Trained	No Level 2 Training	Cycle Training	No Cycle Training
		(*n* = 115)	(*n* = 90)	(*n* = 95)	(*n* = 110)
Age	Mean	12.62	13.20	45.06	43.17
	Median	12.00	13.00	44.50	43.00
	Range	11–17	11–18	32–66	31–63
Gender (*n*)	Female	57	51	71	88
	Male	56	37	24	19
	Non-binary	1	0	0	0
	Prefer not to say	1	2	0	3
Physical Impairment	Yes	1	2	9	12
	No	113	86	84	92
	Prefer not to say	1	2	2	6
Mental Impairment	Yes	4	3	4	3
	No	109	84	87	104
	Prefer not to say	2	3	4	3

### Procedure

Institutional research ethics committee approval was obtained prior to commencing data collection. The survey was circulated with the support of Modeshift, a UK-based sustainable travel organisation whose aim is to “secure increased levels of safe, active and sustainable travel in business, education and community settings”. Modeshift works closely with schools in 15 UK local authorities, whereby students act as *Active Travel Ambassadors* (ATAs), to increase use of active and sustainable travel to their fellow students, and to empower their schools and fellow pupils to tackle congestion, road safety and air quality. Modeshift circulated the survey via 86 of their participating English secondary schools and academies, which were diverse in terms of their geographical locations, their denominations, and student demographics (a list of schools is in the Supplementary Materials). School offices were contacted and were asked to circulate the survey to parents and carers in all year groups.

All participants were provided with an electronic participant information sheet immediately prior to completing the survey, then provided their informed consent via an online form prior to their participation, which included their understanding of their right to withdraw their data, to no personal disadvantage whatsoever, at any time.

On following the QR code link, the parent viewed a welcome message, which stated the approximate survey duration (~ 20 min) and recommended that the parent should read the associated participant information sheet; they were also invited to preview the survey, so they could make an informed decision about whether to proceed. Both documents were available in PDF format via clickable links. Once they had read the participant information sheet and asked any questions of the first author via email if required (no one took this option), the parent completed an online consent form for themself and their child, before the young person and parent completed the survey.

### Materials and measures

A PDF flyer invited parents/carers and their children to take part in a survey study entitled *Young People’s Travel Behaviour*,* Physical Activity*,* and Wellbeing*. The flyer summarised the study and included advertisement of a prize draw for twenty £50 online retailer gift cards; it also explained that the prize draw would take place when the survey ended, and that participants would only be eligible for the draw after parental verification of the authenticity of their survey responses (see Data Analysis). The flyer comprised a QR code that could be scanned using a smartphone camera to access the survey, which was administered via the JISC survey platform (JISC, 2024)[46]. A copy of the full survey is available in Supplementary Materials, and some additional information was collected but the contents relevant to the present study are summarised below in order of their appearance.

#### Demographic information, active travel behaviour and attitudes

Child and parent provided demographic information including their age and gender identity, their Bikeability/cycle training status, and reasons for not completing Bikeability cycle training (child only). Then they stated the frequency with which they made journeys via active (e.g., cycling, walking) and passive (e.g., car, train) travel modes, according to six categories: *Once or more a day*, *Once or more a week*, *Once or more a month*, *Once or more a year*, *Less than once a year*, or *Never*. Child and parent also indicated their attitudes towards cycling via six items, three of which were positively phrased (*cycling is efficient*, *cycling on roads is convenient*, *cycling is relaxing*), three of which were negatively framed (*cycling is tiring*, *cycling on roads is stressful*, *cycling on roads is dangerous*).

#### Moderate-to-Vigorous physical activity (MVPA) levels and sedentariness

Children detailed their levels of MVPA in the week preceding their completion of the survey, via a bespoke measure, which asked them to recall the number of hours for which they were very active, and hours for which they were moderately active, when they were at school, and when they were not at school *in the preceding week*. They were also asked to recall the number of hours they spent sitting or lying down (i.e., sedentary) at school, during their free time on weekdays, and at weekends; this was also a bespoke measure. Examples of vigorous and moderate activities, and sedentary behaviour, were provided to facilitate their estimates. This comparatively short-term recall approach was chosen because people’s retrospective recall of their physical activity tends to be spurious [90]. We did not collect MVPA data from parents for the sake of survey concision, despite evidence that adolescents’ physical activity levels are weakly correlated with those of their parents [81].

#### Subjective wellbeing measures

In line with recent academic discussions about alternative approaches to measuring wellbeing [24, 32, 32], three different but complementary measures, encompassing evaluative, experiential and eudemonic elements [31] were employed. The measures are described below.

#### UK wellbeing measures

We employed eight items based on the United Kingdom’s national wellbeing measures [62]: (1)*“Please rate your overall satisfaction with your life”*, (2) *“Please rate the extent to which you feel the things you do in life are worthwhile”*, (3) *“Please rate how happy you felt yesterday”*, (4) *“Please rate how anxious you felt yesterday”*, (5) *“Please indicate how frequently you feel lonely”*, (6) *“Please indicate the extent to which you agree with the statement, "I can rely on the people in my life if I have a serious problem"”*, (7) *“Please rate how much, in general, you trust most people”* and (8) *“Please rate your satisfaction with your general health”*. Items 1-4 reflect the questions included in the ONS4 (ONS, 2021). Participants responded to all items on scales anchored 0 (zero) to 10, with varying labels (see Supplementary Materials for the full survey).

#### Life satisfaction single-item measure

Responses to the first of the national wellbeing measures, which is a slightly modified version of the ONS question, *“Overall*,* how satisfied are you with your life nowadays?’*,* where answers range from 0 (‘Not at all’) to 10 (‘Completely’)?”*, were analysed separately because of the potential economic relevance of responses to this item [24, 31].

#### The warwick-edinburgh mental wellbeing scale (WEMWBS)

The 14-item WEMWBS [83] is a positively worded measure of subjective wellbeing that has been utilised and validated in a variety of contexts and populations [4, 7, 14, 21, 57, 61].

Responses on The WEMWBS and its 7-item derivative, the SWEMWBS, generally converge on one solitary factor – wellbeing – although recent analysis suggest that the shorter version may be more robust in this regard [74]. Nonetheless, we employed the original measure due to (a) its widespread usage and (b) its balance of items that focus on *feeling* (e.g., *“I’ve been feeling optimistic about the future”*) and *functioning* (*“I’ve had energy to spare”*). Respondents indicate the extent to which they have experienced each state over the preceding two weeks. The authors noted that an increase or decrease of 3 points represents a meaningful change or difference in subjective wellbeing.

#### Parental satisfaction with living circumstances

Because a child’s living circumstances determine their wellbeing [66, 69], we sought to establish whether there were any significant between-group differences in parental satisfaction with their living conditions. Therefore, we asked parents to rate the *levels of crime in their area*, their *feelings of safety when walking alone after dark*, their *satisfaction with their access to green spaces and key services* (e.g., general practitioners), their *sense of community*, their *satisfaction with their place of residence*, and their *satisfaction with their household income*, drawing eight items from the UK national wellbeing measures (see Supplementary Materials). Parents were asked to respond in respect to two different timepoints: (1) at the time of survey completion and (2) when their child was ten years old, the midpoint of the age range during which Bikeability Level 2 cycle training is offered to UK children.

#### Additional data

Some additional survey data were collected, data which have not been subject to analysis in this study. Most of these data are available in the anonymised raw dataset available on Mendeley Data (see Supplementary Materials), anonymisation permitting.

### Data analysis

Of the 276 survey responses received, all were screened for their authenticity, via communication with parents via telephone call and/or email, in which they were required to confirm some of their survey responses. Seventy-one cases for which there was either no reply, or dubious answers given, were discarded.

All continuous data were screened for univariate outliers and tested for normality. Z score analyses, using a cutoff of ± 3, showed that there were two marginal univariate outliers, for *positive attitudes towards cycling* (child [+ 3.11] and parent [3.03]; case 195). However, considering the nature of this measure, which could conceivably comprise data from cycling enthusiasts, we chose to retain these two datapoints. Screening for multivariate outliers, using the Malahanobis Distance test, with threshold probability set at *p* =.001, revealed four outlying cases (2, 70, 72 and 167; see raw data in Supplementary Materials). Visual inspection suggested only one obvious discrepant case, wherein the participant’s scores for the two composite wellbeing measures were noticeably different. However, we opted to remove all four cases from all inferential analyses to maintain statistical integrity (NB: analyses including these cases yielded near-identical results).

Inspection of standardised skewness and kurtosis statistics showed that (i) MVPA values were positively skewed and moderately leptokurtic, (ii) aggregated scores on the national wellbeing measures were negatively skewed, and (iii) scores on the single item wellbeing measure were negatively skewed and strongly leptokurtic. The Kolmogorov-Smirnov test of normality showed violations for all three measures. Hence, nonparametric tests were used.

Between-group comparisons for all three subjective wellbeing measures were analysed using independent samples t tests (or Mann-Whitney U Test for nonparametric data), as were MVPA and sedentariness data, and parental satisfaction with their living circumstances, past and present. Chi squared tests of independence were used to ascertain the extent of relationships between participants’ Bikeability/cycle training status and their active travel behaviour, between child Bikeability cycle training status and their parent’s cycle training status, and between child cycling behaviour and their parent’s cycling behaviour. Follow-up analyses of differences in wellbeing according to active travel frequencies were conducted using one-way ANOVA (WEMWBS) and Kruskal-Wallis ANOVA (national wellbeing and single-item measures).

Relationships between scores on the wellbeing measures were subjected to correlation analyses (Pearson’s *r* or Kendall’s Tau coefficient), as was the relationship between children’s and parents’ attitudes towards cycling. We explored potential between-group differences in attitudes towards cycling, for both children and parents, using independent samples t tests.

## Results

### Subjective wellbeing

**Fig. 1:**
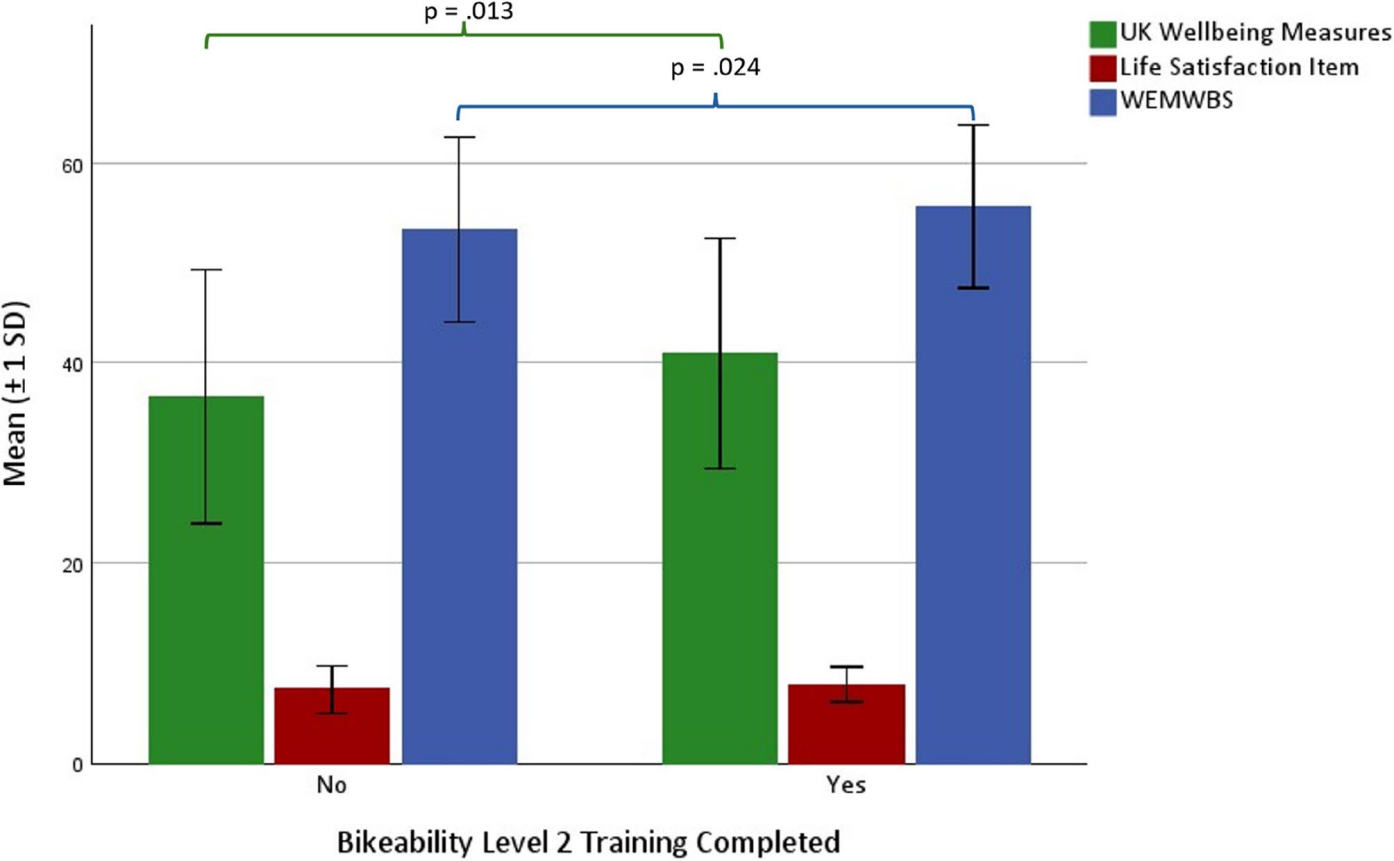
Between-Group Differences in Subjective Wellbeing

Consistent with our predictions, Bikeability Level 2 graduates reported greater subjective wellbeing than children who had not completed Bikeability Level 2 cycle training on the combined UK national wellbeing measures, *U*(201) = 5988.00, *p* =.013, *Z* = 2.49, 95% CI = 0.95–7.63, and on the WEMWBS, *t*(199) = 1.99, *p* =.024, Cohen’s *d* = 0.28, 95% CI = −0.02–4.87. There were no between-group differences on the single-item measure, *p* =.330. Figure 1 shows the means and standard deviations for all three measures.

Participants’ ratings on the UK national wellbeing measures were moderately strongly correlated with those on the WEMWBS, *r*_*τ*_ (201) = 0.57, *p* <.001, and the single-item measure, *r*_*τ*_ (201) = 0.60, *p* <.001. Ratings on the WEMWBS and Life Satisfaction item were also moderately correlated,*r*_*τ*_ (201) = 0.52, *p* <.001.

### Active travel behaviour

There was an association between the children’s Bikeability cycle training status and the frequency with which they made journeys by cycling, χ^2^(5, *N* = 200) = 20.64, *p* <.001, Phi = 0.32, and walking, χ^2^(4, *N* = 200) = 12.07, *p* =.017, Phi = 0.25, despite no between-group differences in attitudes towards cycling, *p*’s > 0.05.

There was also an association between the parents’ training status and the frequency with which they made journeys by cycling, χ^2^(5, *N* = 199) = 12.45, *p* =.029, Phi = 0.25; there was no such association for cycle training and journeys made by walking. Table 2 shows the frequency of cycling and walking journeys made by children and parents, grouped according to Bikeability/cycle training status.

#### Young people’s wellbeing, by active travel frequency

There were differences in WEMWBS scores across the six cycling frequencies (*Never*, *Less than once a year*, *Once or more a year*, *Once or more a month*, *Once or more a week*, *Once or more a day*), *F*(4,34) = 4.11, $\:{\eta\:}_{p}^{2}$ = 0.33, *p* =.008. Bonferroni-corrected follow-up comparisons showed that young people who cycled *once or more a week* (*M* = 60.00, *SD* = 7.45) reported higher wellbeing on the WEMWBS than those who only did so *once or more a year* (*M* = 47.25, *SD* = 6.96), *p* =.010, 95% CI of the difference = 2.11–23.39, and those who *never* cycled (*M* = 47.70, *SD* = 5.58), *p* =.019, 95% CI of the difference = 1.35–23.25.

There were no significant differences on the other wellbeing measures, nor when differences in wellbeing scores were assessed across walking frequencies, all *p*’s > 0.05.

### MVPA and sedentariness

Contrary to our predictions, there were no differences between Bikeability Level 2 graduates’ reported weekly hours of MVPA (*M* = 16.96, *SD* = 0.83) and sedentariness (*M* = 35.97, *SD* = 1.43) and those of their non-graduate counterparts (*M* = 15.28, *SD* = 1.00 and *M* = 35.34, *SD* = 1.96, respectively), *p*’s > 0.05.

### Parental satisfaction with living circumstances

There were no significant between-group differences in parents’ satisfaction with their living circumstances, neither at the time of the survey (*M* = 27.72, *SD* = 0.48 vs. *M* = 26.37, *SD* = 0.58) nor when their child was 10 years old (*M* = 28.80, *SD* = 0.44 vs. *M* = 27.49, *SD* = 0.59), in line with our predictions, *p*’s > 0.05. However, consistent with our expectations, parental satisfaction with their living circumstances at the time of the survey was positively correlated with their children’s subjective wellbeing, for all three wellbeing measures, *r*_*τ*_*’s* (201) = 0.20–0.22, *p* <.001.

### Parents’ and children’s cycling behaviour and attitudes

There was an association between parent cycle training status and child Bikeability cycle training status: 63 of the parents whose children had not completed Bikeability Level 2 cycle training had not completed any cycle training themselves, only 25 had done so, χ^2^(1, *N* = 201) = 10.72, *p* =.001, Phi = 0.23. Differences in parent cycle training status for children who had completed Bikeability Level 2 cycle training were negligible: 55 parents had not completed training, whereas 58 had done so.

There was also an association between parents’ cycle journey frequency and child cycle journey frequency, χ^2^(25, *N* = 199) = 97.78, *p* <.001, Phi = 0.70. Moreover, children’s and parents’ attitudes towards cycling were moderately correlated, *r*(201) = 0.59, *p* <.001. There were no between-group differences in the children’s attitudes towards cycling, *p* >.05, but the parents who had completed cycle training (*M* = 18.02, *SD* = 4.59) exhibited more positive attitudes than those who had not completed cycle training (*M* = 16.81, *SD* = 3.85), *t*(199) = 2.02, *p* =.044, Cohen’s *d* = 0.29, 95% CI = 0.08–0.57.

**Table 2:** Children’sand parents’ cyclingability and Behaviour, bybikeability/cycle training

	Child (*n* [%])		Parent (*n* [%])	
	Bikeability Level 2 Trained	No Level 2 Training	Cycle Training	No Cycle Training
*Please indicate how frequently you make journeys by cycling (incl. e-cycles)*				
Once or more a day	19 [16.5]	7 [7.9]	1 [1.2]	3 [2.5]
Once or more a week	29 [25.2]	10 [11.2%]	13 [13.3]	6 [5.8]
Once or more a month	26 [22.6]	17 [19.1]	18 [19.3]	5 [5.0]
Once or more a year	20 [17.4]	13 [14.6]	23 [24.1]	22 [20.0]
Less than once a year	8 [7.0]	12 [13.5]	6 [6.0]	13 [11.7]
Never	13 [11.3]	30 [33.7]	34 [36.1]	60 [55.0]
*Please indicate how frequently you make journeys by walking*				
Once or more a day	86 [74.8]	61 [68.5]	65 [68.7]	73 [67.3]
Once or more a week	25 [21.7]	14 [15.7]	20 [20.5]	20 [18.0]
Once or more a month	2 [1.7]	9 [10.1]	8 [8.4]	8 [7.4]
Once or more a year	0 [0.0]	3 [3.4]	1 [1.2]	3 [2.5]
Less than once a year	0 [0.0]	0 [0.0]	0 [0.0]	0 [0.0]
Never	2 [1.7]	2 [2.2]	1 [1.2]	5 [4.8]

Data missing for one child and one parent (both no Bikeability/cycle training)

## Discussion

We circulated an online survey to parents and carers (hereafter abbreviated to ‘parents’) of young people in UK secondary schools. Two hundred-and-one young people contributed data to questions pertaining to their subjective wellbeing, physical activity, sedentariness, active travel behaviour, attitudes towards cycling, and Bikeability Level 2 cycle training status (i.e., Yes/No). Their parents reported their active travel behaviour, cycle training status, and attitudes towards cycling. Additionally, parents reported their satisfaction with their living circumstances, both present and past.

Our hypotheses were partly supported: Level 2 graduates reported greater subjective wellbeing than their non-graduate peers, approximately three years, on average, after completing the training – and parents’ reports of their current or past living circumstances did not differ between groups. However, there were also no differences in the groups’ MVPA and sedentariness – although active travel behaviour did differ: Level 2 graduates walked and cycled more frequently and reported more positive attitudes towards cycling. Similarly, parents who had completed Bikeability cycle training cycled more than those who did not. In support of our predictions, there were positive associations between the young people’s and their parents’ active travel behaviour, cycle training status and cycling attitudes. Additionally, more frequent use of cycling for active travel was associated with greater subjective wellbeing.

To our knowledge, this is the first time that scores on the UK national wellbeing measures have been reconciled with scores on the WEMWBS. It is encouraging that differences in self-reported subjective wellbeing emerged between the two groups, on both the national wellbeing measures and the WEMWBS, and that the mean difference in the two groups’ WEMWBS scores (2.43 points) was comparable to those previously observed for hypnotherapy sessions to treat anxiety and depression (SWEMWBS; [77]). Moreover, the scores on these measures were moderately correlated with one another, which suggests that they measure a similar construct, in this case, *wellbeing*. However, differences between groups on the single-item measure did not attain statistical significance, which is disappointing, given the utilisation of this measure for governmental policy decision-making [24, 32].

Contrary to our expectations, MVPA did not differ between groups. This may reflect the inherent difficulty in accurately reporting one’s physical activity levels [90], or that participants’ physical activity in the week preceding their survey completion was not indicative of their overall activity levels. For example, the survey was open during the UK summer vacation period, when schools are closed and so the young people could not report their school-based physical activity levels as required. However, Bikeability Level 2 graduates were more frequent active travellers. The potential benefits of active travel may extend beyond increases in physical activity levels, including increases in economic prosperity, and environmental benefits such as reduced air and noise pollution [30].

There are also benefits of active travel for the individual, such as the increased freedom and flexibility it affords. For example, Orsini and O’Brien [65], who found that teenagers who regularly cycled to school stated that doing so gave them independence and self-empowerment; this is consistent with our need for autonomy [72], and the eudemonic approach to measuring wellbeing [31]. Subsequently, Bjørnarå and colleagues [16] conducted a series of focus groups with 36 parents of young children, to find out more about the factors that influenced their motivation to cycle to work, kindergarten or the grocery store. Like Orsini and O’Brien’s participants, the parents referred to the freedom and flexibility that cycling afforded them, relative to travelling in a car (e.g., *“…it doesn’t mean 12 minutes of waiting. Like it can mean when taking the bus. Or when being stuck in a traffic jam”* [a father of three]). However, there are many barriers to children’s cycling, such as journey distance [59] and parents’ attitudes towards cycling [11]. Relatedly, our data show that the parents’ attitudes and those of their children were correlated, as was their active travel behaviour. Plus, recent data from over 4,000 children and young people show that Bikeability Level 2 cycle training graduates are more likely to cycle to get somewhere, and to cycle for fun, than their untrained counterparts (Bishop et al., unpublished observations). Hence, Bikeability cycle training may transcend such barriers.

### Study limitations

There are several limitations to the present study. The foremost of these is its retrospective design, which limits our ability to infer causality regarding the effect of Bikeability Level 2 cycle training on young people’s wellbeing and active travel patterns. Another is that our MVPA measure might not have reflected the participants’ physical activity levels, because (a) their responses were made in relation to the week preceding survey completion and (b) retrospective recall is flawed. Although our aim in using these items was to minimise errors in people’s recollection of their physical activity levels [90], this might, in hindsight, have impoverished the quality of these data, and so it would have been prudent to ask the participants to indicate how typical, in terms of MVPA, the week preceding the survey was. Moreover, our measures of MPVA and sedentariness have not been validated, and so they might have yielded inaccurate estimates. That said, measurement of physical activity is consistently fraught with error and discrepancy, as illustrated in objective data [56] and self-reports [51]. It is also possible that young people reported their active travel behaviour more consistently, potentially making it a more reliable proxy for their physical activity. This notion should be explored.

Given the profound impact of household circumstances on young people’s wellbeing [40, 66, 69], it was encouraging to see that parental satisfaction with these circumstances did not differ significantly between the two groups. However, we did not collect data regarding other potential influences on living circumstances that ostensibly affect young people’s life satisfaction, such as caring responsibilities [50], nor their satisfaction with their appearance and their school environment, both of which are among the highest causes of dissatisfaction amongst young people in the UK [84]. Relatedly, we could also have posed the same questions to the young people regarding their living conditions – but given their likely unawareness of aspects such as local crime levels and household income when they were 10 years old, we decided to only pose the related questions to their parents.

One other limitation is the sample size. We had planned to collect data from one thousand participants, so that we could employ more sophisticated multivariate techniques to analyse the data in a more nuanced way. However, participant recruitment was challenging, even with support from Modeshift colleagues. One potential explanation for this is that, for the sake of transparency, participants were able to view an entire PDF copy of the survey prior to providing their informed consent, which might have been off-putting for many individuals; the expansion of response scales for each-and-every item might have made the survey appear longer. Nonetheless, this is another feature that we would not change in future, because such transparency is not only ethically appropriate, but also a potential determinant of data quality: those who chose to complete the survey were more likely to be motivated to do so and therefore more likely to complete it assiduously.

### Future research directions

If we are to determine whether Bikeability Level 2 cycle training is truly an economically viable behaviour change intervention – one that leads to increased active travel and greater subjective wellbeing – then prospective longitudinal studies, in which Bikeability training is introduced as an intervention after a substantive baseline period, ideally with follow-up data collection several years later, are essential. One challenge to doing so is the relatively short-term nature of governmental funding for the Bikeability programme: at the time of writing, the programme could theoretically cease to exist within several months. Longer term government investment is required, alongside a commitment to funding prospective longitudinal research that would enable us to determine the true impact of Bikeability training. This would be facilitated by incorporating Bikeability into the UK National Curriculum, just as swimming is presently. The Water Safety (Curriculum) Bill [88] states the rationale for swimming’s inclusion on the curriculum – to save lives – but the number of child fatalities on roads is almost certainly greater: on average, 278 children and young people were killed on UK roads each year between 2016 and 2020 [27], compared to approximately 40 water-related fatalities. Bikeability cycle training is an intervention that may not only improve young people’s lives but also save them.

Another useful next research step would be to understand *why* Bikeability Level 2 graduates might report higher levels of subjective wellbeing and active travel. For example, it would be enlightening to understand those individuals’ perceptions of the advantages of being able to cycle competently and confidently as they become adolescents. If increased active travel does not contribute significantly to the young person’s physical activity levels, then other mechanisms must be explored. For example, it is conceivable that Bikeability cycle training achieves its aim, of empowering the young person to cycle confidently and competently on roads, thereby increasing the distance from home that they can travel quickly and easily, without reliance on third parties, be they parents/carers, bus drivers, or otherwise. This, in turn, may increase the young person’s social network, their employment opportunities, and their independence, satisfying their need to feel autonomous, competent and connected to others [72] – all key components of a eudemonic approach to measuring subjective wellbeing [31].

It would also be worthwhile to examine the impact of Bikeability cycle training on young people’s views of the risks associated with cycling on roads. The Biopsychosocial Model of Challenge and Threat (BPM-CT; [15]) provides a useful framework for doing so. The BPM-CT posits that an individual may perceive a stressful situation as either a *challenge* (a positive affective state) or as a *threat* (a negative one). A recent review [36] shows that, for 62 studies comprising more than 7,000 participants, being in a challenge state leads to superior performance in a variety of domains, including sport and education. Hence, an important feature of Bikeability cycle training may be the development of *challenge state mindsets* in children and young people. Indeed, Bikeability cycle training instructors are encouraged to refrain from use of the words ‘safe’ and ‘safety’ in their delivery, to avoid cultivating a threat state when cycling on roads, and towards other road users. Gamified approaches to cycle training [10, 12, 13] may also be useful in this regard.

## Conclusion

Although it would be imprudent for us to suggest that completing Bikeability Level 2 cycle training as a child might *lead to* greater wellbeing as a young person, our data suggest that Bikeability Level 2 graduates are *more likely* than non-graduates to report greater subjective wellbeing, and more likely to cycle and walk to make journeys – a cooccurrence that may not be coincidence. Given the multiple benefits that active travel may contribute to a thriving economy, be it a financially- or wellbeing-oriented one, it behoves us to further investigate why Bikeability cycle training might be a route to increasing young people’s subjective wellbeing.

## Supplementary Information

Supplementary Material 1.

Supplementary Material 2.

Supplementary Material 3.

Supplementary Material 4.

Supplementary Material 5.

## Data Availability

Data are provided within the supplementary information files, as well as in Mendeley Data, at the following 10.17632/tp6msdmwm9.1.

## References

[1] Aldred R, Croft J, Goodman A. Impacts of an active travel intervention with a cycling focus in a suburban context: One-year findings from an evaluation of london’s in-progress mini-Hollands programme. Transp Res Part A: Policy Pract. 2019;123:147–69. 10.1016/j.tra.2018.05.018.

[2] Aldred R, Watson T, Lovelace R, Woodcock J. Barriers to investing in cycling: stakeholder views from England. Transp Res Part A: Policy Pract. 2017;128:149–59. 10.1016/j.tra.2017.11.003. 10.1016/j.tra.2017.11.003 PMC6703189 31582879

[3] Aldred R, Woodcock J, Goodman A. Major investment in active travel in outer london: impacts on travel behaviour, physical activity, and health. J Transp Health. 2021;20. 10.1016/j.jth.2020.100958.

[4] Aldubaybi AA, Coneyworth LJ, Jethwa PH. Assessing the prevalence and potential drivers of food insecurity and the relationship with mental wellbeing in UK university students: a cross-sectional study. Nutr Bull. 2024;49(1):96–107. 10.1111/nbu.12662. 38311588 10.1111/nbu.12662

[5] Alhroub N, Al-Sarairhe I, Awamleh RA, Ayasreh I, Alkhawaldeh A, ALBashtawy M, Oweidat IA, ALBashtawy S, Ayed A, ALBashtawy Z, Abdalrahim A, Alkhawaldeh H. Physical activity barriers among adolescents in jordan: A Cross-Sectional study. SAGE Open Nurs. 2024;10:23779608241272690. 10.1177/23779608241272688. 10.1177/23779608241272688 PMC11334122 39165912

[6] Alliott O, Fairbrother H, van Sluijs E. Adolescents’ physical activity during and beyond the Covid-19 pandemic: a qualitative study exploring the experiences of young people living in the context of socioeconomic deprivation. BMC Public Health. 2024;24(1):2450. 10.1186/s12889-024-19777-z. 39434034 10.1186/s12889-024-19777-z PMC11494794

[7] Alsarrani A, Hunter R, Dunne L, Garcia L. Association between friendship quality and subjective wellbeing in adolescents: a cross-sectional observational study. Lancet (London England). 2023;402(Suppl 1):S21. 10.1016/S0140-6736(23)02108-6. https://doi-org.ezproxy.brunel.ac.uk/. 37997061 10.1016/S0140-6736(23)02108-6

[8] Arvidsen J, Schmidt T, Præstholm S, et al. Demographic, social, and environmental factors predicting danish children’s greenspace use. Urban Forestry Urban Green. 2022;69(NPAG). 10.1016/j.ufug.2022.127487.

[9] Bell SL, Audrey S, Gunnell D, Cooper A, Campbell R. The relationship between physical activity, mental wellbeing and symptoms of mental health disorder in adolescents: a cohort study. Int J Behav Nutr Phys Act. 2019. 10.1186/s12966-019-0901-7. 31878935 10.1186/s12966-019-0901-7 PMC6933715

[10] Bishop D, Broadbent D, Daylamani-Zad D, Fukaya K, Smith BR. 'Can Immersive Training Complement On-Road Cycle Training for Children? Two Intervention Studies in Urban and Rural UK Communities'. J Transp Health. 2025;42:102048–102048. 10.1016/j.jth.2025.102048; https://www.sciencedirect.com/science/article/pii/S2214140525000684.

[11] Bishop DT, Batley P, Waheed H, Dkaidek TS, Atanasova G, Broadbent DP. Barriers and enablers for cycling: a COM-B survey study of UK schoolchildren and their parents. J Transp Health. 2024;35: 101765. 10.1016/j.jth.2024.101765.

[12] Bishop DT, Daylamani-Zad D, Dkaidek TS, Fukaya K, Broadbent DP. A brief gamified immersive intervention to improve 11–14-year-olds’ cycling-related looking behaviour and situation awareness: a school-based pilot study. Transp Res Part F Traffic Psychol Behav. 2023;97:17–30. 10.1016/j.trf.2023.06.019.

[13] Bishop DT, Engelhard P, Fukaya K, Dkaidek TS, Sassoon I. Classroom-based delivery of immersive cycle training to upskill and enthuse children and young people: A tool to transcend socioeconomic barriers. Manuscript in preparation; 2025b.

[14] Blake H, Mancini H, Coyne E, Cooper J, Stanulewicz-Buckley N. Workforce wellbeing centres and their positive role for wellbeing and presenteeism in healthcare workers during the COVID-19 pandemic: secondary analysis of COVID-Well data. BMC Health Serv Res. 2024;24(1):302. 10.1186/s12913-024-10730-9. https://doi-org.ezproxy.brunel.ac.uk/. 38448919 10.1186/s12913-024-10730-9 PMC10918935

[15] Blascovich J, Mendes WB. Challenge and threat appraisals: The role of affective cues. In: Forgas JP, ed. Feeling and thinking: The role of affect in social cognition. Cambridge University Press; 2000. p. 59–82. https://psycnet.apa.org/record/2000-07085-002.

[16] Bjørnarå HB, Westergren T, Fegran L, Te Velde SJ, Fyhri A, Deforche B, Andersen LB, Berntsen S, Bere E. Cumbersome but desirable-Breaking the code of everyday cycling. PLoS ONE. 2020;15(9):e0239127. 10.1371/journal.pone.0239127. 32925959 10.1371/journal.pone.0239127 PMC7489513

[17] Bourke M, Hilland TA, Craike M. Daily physical activity and satisfaction with life in adolescents: an ecological momentary assessment study exploring direct associations and the mediating role of core affect. J Happiness Stud. 2022;23(3):949–68. 10.1007/s10902-021-00431-z.

[18] Buehler R, Kuhnimhof T, Bauman A, Eisenmann C. Active travel as stable source of physical activity for one third of German adults: evidence from longitudinal data. Transp Res Part A Policy Pract. 2019;123:105–18. 10.1016/j.tra.2018.09.022.

[19] Çakir G, Isik U, Kavalci İ. An evaluation of physical activity levels and mental health among young people: a cross-sectional study. BMC Psychol. 2025;13(1):204. 10.1186/s40359-025-02533-2. 40045417 10.1186/s40359-025-02533-2 PMC11884174

[20] Caldwell DM, Davies SR, Hetrick SE, Palmer JC, Caro P, López-López JA, Gunnell D, Kidger J, Thomas J, French C, Stockings E, Campbell R, Welton NJ. School-based interventions to prevent anxiety and depression in children and young people: a systematic review and network meta-analysis. Lancet Psychiatry. 2019;6(12):1011–20. 10.1016/S2215-0366(19)30403-1. 31734106 10.1016/S2215-0366(19)30403-1 PMC7029281

[21] Carver A, Rachele JN, Sugiyama T, Corti B-G, Burton NW, Turrell G. Public greenspace and mental wellbeing among mid-older aged adults: findings from the HABITAT longitudinal study. Health Place. 2024;89:103311.. doi-org.ezproxy.brunel.ac.uk/10.1016/j.healthplace.2024.103311. 39032205 10.1016/j.healthplace.2024.103311

[22] Caspersen CJ, Powell KE, Christenson GM. Physical activity, exercise, and physical fitness: definitions and distinctions for health-related research. Public Health Rep (Washington D C: 1974). 1985;100(2):126–31. PMC1424733 3920711

[23] Connell H, Logan G, Somers C, Baker G, Broadfield S, Bunn C, Harper LD, Kelly P, McIntosh E, Pell JP, Puttnam J, Robson S, Gill JMR, Gray CM. Development and optimisation of a multi-component workplace intervention to increase cycling for the cycle Nation project. Front Sports Act Living. 2022;4:857554. 10.3389/fspor.2022.857554. 36385778 10.3389/fspor.2022.857554 PMC9643150

[24] Cooper K, Fabian M, Krekel C. New approaches to measuring welfare. Fisc Stud. 2023;44(2):123–35. 10.1111/1475-5890.12333.

[25] Cummings A, Shelton K. The prevalence of mental health disorders amongst care-experienced young people in the UK: a systematic review. Child Youth Serv Rev. 2024;156:1–11. 10.1016/j.childyouth.2023.107367.

[26] Department for Transport. National Standard for Cycle Training. 2019. https://www.gov.uk/government/publications/national-standard-for-cycle-training.

[27] Department of Health & Social Care. Fatal casualties from road traffic accidents (aged 0–24). Fingertips Public Health Profiles. 2021. Retrieved from 2025. https://fingertips.phe.org.uk/profiles.

[28] Diener E. Subjective well-being. Psychol Bull. 1984;95(3):542–75. 6399758

[29] Diener E, Lucas RE, Oishi S. Subjective well-being: The science of happiness and life satisfaction. In: Snyder CR, Lopez SJ, eds. Handbook of positive psychology. Oxford University Press; 2002. p. 463–73. https://psycnet.apa.org/record/2002-02382-005.

[30] Ding D, Luo M, Infante MFP, Gunn L, Salvo D, Zapata-Diomedi B, Smith B, Bellew W, Bauman A, Nau T, Nguyen B. The co-benefits of active travel interventions beyond physical activity: a systematic review. Lancet Planet Health. 2024;8(10):e790–803. 10.1016/S2542-5196(24)00201-8. 39393380 10.1016/S2542-5196(24)00201-8

[31] Dolan P, Metcalfe R. Measuring subjective wellbeing: recommendations on measures for use by national governments. J Soc Policy. 2012;41(2):409–27. 10.1017/S0047279411000833.

[32] Frijters P, Clark A, Krekel C, Krekel C. A happy choice: wellbeing as the goal of government. Behav Public Policy. 2020;4(2):126–65. 10.1017/bpp.2019.39.

[33] Furjes-Crawshaw J, Heke I, Jowett T, Rehrer NJ. The physical activity environment, nature-relatedness and wellbeing. Int J Environ Res Public Health. 2025;22(2): 299. 10.3390/ijerph22020299. 40003524 10.3390/ijerph22020299 PMC11855637

[34] Garcia L, Mendonça G, Benedetti TRB, Borges LJ, Streit IA, Christofoletti M, Silva-Júnior FLe, Papini CB, Binotto MA. Barriers and facilitators of domain-specific physical activity: a systematic review of reviews. BMC Public Health. 2022;22(1):1–22. 10.1186/s12889-022-14385-1. 36289461 10.1186/s12889-022-14385-1 PMC9598005

[35] Gennings E, Brown HJ, Hewlett D, Batten J. Children and young people’s perspectives from UK lockdown: leisure-less experiences. Leis Stud. 2023;42(1):147–55. 10.1080/02614367.2022.2107052.

[36] Hase A, Nietschke M, Kloskowski M, Szymanski K, Moore L, Jamieson JP, Behnke M. The effects of challenge and threat States on performance outcomes: an updated review and meta-analysis of recent findings. EXCLI J. 2025;24:151–76. 10.17179/excli2024-7995. 40027878 10.17179/excli2024-7995 PMC11869992

[37] Hazir SG, Ryan C, Moore A, Lewis C, Lunn J. The role of the multiple index of deprivation in predicting mental health outcomes after the COVID-19 pandemic in adolescents: a cross-sectional study. Lancet. 2023;402:S47. 10.1016/S0140-6736(23)02143-8. 37997089 10.1016/S0140-6736(23)02143-8

[38] HM Treasury. Wellbeing discussion paper: monetisation of life satisfaction effect sizes. A review of approaches and proposed approach. 2021. https://assets.publishing.service.gov.uk/media/60fa91c68fa8f50431ca80da/Wellbeing_guidance_for_appraisal_-_background_paper_reviewing_methods_and_approaches.pdf.

[39] Hoare E, Milton K, Foster C, Allender S. The associations between sedentary behaviour and mental health among adolescents: a systematic review. Int J Behav Nutr Phys Act. 2016;13:1–22. 10.1186/s12966-016-0432-4. 27717387 10.1186/s12966-016-0432-4 PMC5055671

[40] Hock ES, Blank L, Fairbrother H, Clowes M, Cuevas DC, Booth A, Clair A, Goyder E. Exploring the impact of housing insecurity on the health and wellbeing of children and young people in the United Kingdom: a qualitative systematic review. BMC Public Health. 2024;24(1):2453. 10.1186/s12889-024-19735-9. 39251944 10.1186/s12889-024-19735-9 PMC11385840

[41] Hodgson C, Worth J. Research into the impact of bikeability cycle training on children’s ability to perceive and appropriately respond to hazards when cycling on the road. Slough: NFER; 2015.

[42] Holmes E, Arkesteijn M, Knowles K, McKinney T, Mizen A, Purcell C. Understanding the interactions that children and young people have with their natural and built environments: a survey to identify targets for active travel behaviour change in Wales. PLoS One. 2024;19(10):1–16. 10.1371/journal.pone.0311498. 10.1371/journal.pone.0311498 PMC11488727 39423197

[43] Huang J-H, Hipp JA, Marquet O, Alberico C, Fry D, Mazak E, Lovasi GS, Robinson WR, Floyd MF. Neighborhood characteristics associated with park use and park-based physical activity among children in low-income diverse neighborhoods in new York City. Prev Medicine: Int J Devoted Pract Theory. 2020;131. 10.1016/j.ypmed.2019.105948. 10.1016/j.ypmed.2019.105948 31836479

[44] Ibáñez Román JE, Ekholm O, Algren MH, Koyanagi A, Stewart-Brown S, Hall EE, Stubbs B, Koushede V, Thygesen LC, Santini ZI. Mental wellbeing and physical activity levels: a prospective cohort study. Ment Health Phys Act. 2023;24:1–9. 10.1016/j.mhpa.2022.100498.

[45] Ikeda E, Guagliano JM, Atkin AJ, Sherar LB, Ekelund U, Hansen B, Northstone K, van Sluijs E, On behalf of the International Children’s Accelerometry Database (ICAD) Collaborators, Salmon, Riddoch J, Judge C, Cooper K, Griew A, Andersen P, Anderssen LB, Cardon S, Davey G, Hallal R, P., Jago R. Cross-sectional and longitudinal associations of active travel, organised sport and physical education with accelerometer-assessed moderate-to-vigorous physical activity in young people: the International Children’s Accelerometry Database. Internaal J Behav Nut & Phys Act. 2022;19(1), 1–12. 10.1186/s12966-022-01282-4. 10.1186/s12966-022-01282-4 PMC8977036 35366914

[46] Jisc Online Surveys. Jisc. Bristol, UK; 2024.

[47] Kelly P, Bourne J, Richards J, Salvo D, Gill JMR. Editorial: walking, cycling and active travel as part of physical activity and public health systems. Front Sports Act Living. 2023;5:1321450. 10.3389/fspor.2023.1321450. 38022780 10.3389/fspor.2023.1321450 PMC10646603

[48] Kimm SYS, Glynn NW, McMahon RP, Voorhees CC, Striegel-Moore RH, Daniels SR. Self-perceived barriers to activity participation among sedentary adolescent girls. Med Sci Sports Exerc. 2006;38(3):534–40. 10.1249/01.mss.0000189316.71784.dc. 16540842 10.1249/01.mss.0000189316.71784.dc

[49] Koch LC, Lunsky Y, St John L. Physical activity, sedentary behaviour, sleep and mental wellbeing in family caregivers of adults with intellectual and/or developmental disabilities. J Appl Res Intellect Disabilities: JARID. 2025;38(1):e13310. 10.1111/jar.13310. 10.1111/jar.13310 39444261

[50] Lacey RE, Letelier A, Xue B, McMunn A. Changes in life satisfaction, self-esteem, and self-rated health before, during, and after becoming a young carer in the UK: a longitudinal, propensity score analysis. Lancet Reg Health Europe. 2024;50:101187. 10.1016/j.lanepe.2024.101187. 39810990 10.1016/j.lanepe.2024.101187 PMC11726805

[51] Liangruenrom N, Suttikasem K, Widyastari DA, Potharin D, Katewongsa P. Reliability and validity of time-use surveys in assessing 24-hour movement behaviors in adults. J Exerc Sci Fit. 2025;23(2):133–40. 10.1016/j.jesf.2025.03.003. 40225047 10.1016/j.jesf.2025.03.003 PMC11986218

[52] Macintyre S. Deprivation amplification revisited; or, is it always true that poorer places have poorer access to resources for healthy diets and physical activity? Int J Behav Nutr Phys Act. 2007;4:32. 10.1186/1479-5868-4-32. 17683624 10.1186/1479-5868-4-32 PMC1976614

[53] Macintyre S, Macdonald L, Ellaway A. Do poorer people have poorer access to local resources and facilities? The distribution of local resources by area deprivation in Glasgow. Scotl Social Sci Med. 2008;67(6):900–14. 10.1016/j.socscimed.2008.05.029 PMC2570170 18599170

[54] Malnes L, Berntsen S, Kolle E, Ivarsson A, Dyrstad SM, Resaland GK, Solberg R, Haugen T. School-based physical activity in relation to active travel– a cluster randomized controlled trial among adolescents enrolled in the school in motion study in Norway. Int J Behav Nutr Phys Act. 2023;20(1):1–8. 10.1186/s12966-023-01534-x. 37990252 10.1186/s12966-023-01534-x PMC10664674

[55] McCartney G, Hensher M, Trebeck K. How to measure progress towards a wellbeing economy: distinguishing genuine advances from window dressing. Public Health Res Pract. 2023;33(2). 10.17061/phrp3322309. 10.17061/phrp3322309 37406651

[56] McCrorie P, Mitchell R, Ellaway A. Comparison of two methods to assess physical activity prevalence in children: an observational study using a nationally representative sample of Scottish children aged 10–11 years. BMJ Open. 2018;8(1):e018369. 10.1136/bmjopen-2017-018369. 29371272 10.1136/bmjopen-2017-018369 PMC5786112

[57] Melendez-Torres GJ, Hewitt G, Hallingberg B, Anthony R, Collishaw S, Hall J, Murphy S, Moore G. Measurement invariance properties and external construct validity of the short Warwick-Edinburgh mental wellbeing scale in a large national sample of secondary school students in Wales. Health Qual Life Outcomes. 2019. 10.1186/s12955-019-1204-z. 10.1186/s12955-019-1204-z PMC6694652 31412878

[58] Moore GF, Anthony RE, Hawkins J, Van Godwin J, Murphy S, Hewitt G, Melendez TGJ. Socioeconomic status, mental wellbeing and transition to secondary school: analysis of the school health research network/health behaviour in school-aged children survey in Wales. Br Edu Res J. 2020;46(5):1111–30. 10.1002/berj.3616. 10.1002/berj.3616 PMC7818461 33518839

[59] Nelson NM, Foley E, O'Gorman DJ, Moyna NM, Woods CB. Active commuting to school: how far is too far?. Int J Behav Nutr Phys Act. 2008;5:1. 10.1186/1479-5868-5-1; https://pubmed.ncbi.nlm.nih.gov/18182102/. 10.1186/1479-5868-5-1 PMC2268942 18182102

[60] NHS England. Mental Health of Children and Young People in England, 2020: Wave 1 follow up to the 2017 survey. 2020. https://digital.nhs.uk/data-and-information/publications/statistical/mental-health-of-children-and-young-people-in-england/2020-wave-1-follow-up.

[61] Ng Fat L, Scholes S, Boniface S, Mindell J, Stewart-Brown S. Evaluating and Establishing National norms for mental wellbeing using the short Warwick-Edinburgh mental Well-being scale (SWEMWBS): findings from the health survey for England. Qual Life Research: Int J Qual Life Aspects Treat Care Rehabilitation. 2017;26(5):1129–44. 10.1007/s11136-016-1454-8. 10.1007/s11136-016-1454-8 PMC5376387 27853963

[62] Office for National Statistics. Measuring national well-being: domains and measures. Retrieved from 2023. https://www.ons.gov.uk/peoplepopulationandcommunity/wellbeing/datasets/measuringnationalwellbeingdomainsandmeasures. 20th February 2025.

[63] Office for National Statistics. Personal well-being user guidance. Retrieved from 2025. https://www.ons.gov.uk/peoplepopulationandcommunity/wellbeing/methodologies/personalwellbeingsurveyuserguide. 20thFebruary 2025.

[64] Office for National Statistics. Young people’s well-being in the UK: 2020. 2021. Retrieved from 2015. https://www.ons.gov.uk/peoplepopulationandcommunity/wellbeing/bulletins/youngpeopleswellbeingintheuk/2020.

[65] Orsini AF, O’Brien C. Fun, fast and fit: influences and motivators for teenagers who cycle to school. Child Youth Environ. 2006;16(1):121. 10.1353/cye.2006.0029.

[66] Parra-Mujica F, Johnson E, Reed H, Cookson R, Johnson M. Understanding the relationship between income and mental health among 16- to 24-year-olds: analysis of 10 waves (2009–2020) of Understanding society to enable modelling of income interventions. PLoS ONE. 2023;18(2):1–22. 10.1371/journal.pone.0279845. 10.1371/journal.pone.0279845 PMC9974116 36854025

[67] Pitchforth J, Fahy K, Ford T, Wolpert M, Viner RM, Hargreaves DS. Mental health and well-being trends among children and young people in the UK, 1995–2014: analysis of repeated cross-sectional National health surveys. Psychol Med. 2019;49(8):1275–85. 10.1017/S0033291718001757. 30201061 10.1017/S0033291718001757 PMC6518382

[68] Public Health England. Social determinants of health. 2017. https://www.gov.uk/government/publications/health-profile-for-england/chapter-6-social-determinants-of-health#living-standards.

[69] Reed HR, Nettle D, Parra-Mujica F, Stark G, Wilkinson R, Johnson MT, Johnson EA. Examining the relationship between income and both mental and physical health among adults in the UK: analysis of 12 waves (2009–2022) of Understanding society. PLoS ONE. 2025;20(3):1–29. 10.1371/journal.pone.0316792. 10.1371/journal.pone.0316792 PMC11884696 40048442

[70] Robinson SA, Bisson AN, Lachman ME, Hughes ML, Ebert J. Time for change: using implementation intentions to promote physical activity in a randomised pilot trial. Psychol Health. 2019;34(2):232–54. 10.1080/08870446.2018.1539487. 30596272 10.1080/08870446.2018.1539487 PMC6440859

[71] Russell JA. Core affect and the psychological construction of emotion. Psychol Rev. 2003;110(1):145–72. 10.1037/0033-295x.110.1.145. 12529060 10.1037/0033-295x.110.1.145

[72] Ryan RM, Deci EL. Self-determination theory: basic psychological needs in motivation, development, and wellness. The Guilford Press; 2017. 10.1521/978.14625/28806.

[73] Salway R, Emm-Collison L, Sebire SJ, Thompson JL, Lawlor DA, Jago R. The association of school-related active travel and active after-school clubs with children’s physical activity: a cross-sectional study in 11-year-old UK children. Int J Behav Nutr Phys Act. 2019;16(1):72. 10.1186/s12966-019-0832-3. 31438985 10.1186/s12966-019-0832-3 PMC6704690

[74] Sarasjärvi KK, Elovainio M, Appelqvist-Schmidlechner K, Solin P, Tamminen N, Therman S. Exploring the structure and psychometric properties of the Warwick-Edinburgh mental Well-Being scale (WEMWBS) in a representative adult population sample. Psychiatry Res. 2023. 10.1016/j.psychres.2023.115465. https://doi-org.ezproxy.brunel.ac.uk/. 328, N.PAG. 37708805 10.1016/j.psychres.2023.115465

[75] Sawka KJ, McCormack GR, Nettel-Aguirre A, Blackstaffe A, Perry R, Hawe P. Associations between aspects of friendship networks, physical activity, and sedentary behaviour among adolescents. J Obesity, 2014;632689. 10.1155/2014/632689. 10.1155/2014/632689 PMC4190696 25328690

[76] Schalkwijk AAH, Bot SDM, de Vries L, Westerman MJ, Nijpels G, Elders PJM. Perspectives of obese children and their parents on lifestyle behavior change: a qualitative study. Int J Behav Nutr Phys Act. 2015;12:102. 10.1186/s12966-015-0263-8. 26283232 10.1186/s12966-015-0263-8 PMC4539727

[77] Shah N, Cader M, Andrews WP, Wijesekera D, Stewart-Brown SL. Responsiveness of the short Warwick Edinburgh mental Well-Being scale (SWEMWBS): evaluation a clinical sample. Health Qual Life Outcomes. 2018. 10.1186/s12955-018-1060-2. 30577856 10.1186/s12955-018-1060-2 PMC6303870

[78] Smith L, Aggio D, Hamer M. Active travel to non-school destinations but not to school is associated with higher physical activity levels in an ethnically diverse sample of inner-city schoolchildren. BMC Public Health. 2017;17(1):1–6. 10.1186/s12889-016-3920-1. 28056909 10.1186/s12889-016-3920-1 PMC5216598

[79] Sport England. Active Lives Children and Young People Survey: Academic Year 2024;2023-24. https://www.sportengland.org/research-and-data/data/active-lives.

[80] Stark J, Meschik M, Singleton PA, Schützhofer B. Active school travel, attitudes and psychological well-being of children. Transp Res Part F Traffic Psychol Behav. 2018;56:453–65. 10.1016/j.trf.2018.05.007.

[81] Su DLY, Tang TCW, Chung JSK, Lee ASY, Capio CM, Chan DKC. Parental Influence on Child and Adolescent Physical Activity Level: A Meta-Analysis. Int J Environ Res Public Health. 2022;19(24):16861. 10.3390/ijerph192416861; https://pmc.ncbi.nlm.nih.gov/articles/PMC9778652/. 10.3390/ijerph192416861 PMC9778652 36554746

[82] Tappe KA, Glanz K, Sallis JF, Zhou C, Saelens BE. Children’s physical activity and parents’ perception of the neighborhood environment: neighborhood impact on kids study. Int J Behav Nutr Phys Act. 2013;10:39–48. 10.1186/1479-5868-10-39. 23531282 10.1186/1479-5868-10-39 PMC3615958

[83] Tennant R, Hiller L, Fishwick R, Platt S, Joseph S, Weich S, Parkinson J, Secker J, Stewart-Brown S. The Warwick-Edinburgh mental well-being scale (WEMWBS): development and UK validation. Health Qual Life Outcomes. 2007;5:63. 10.1186/1477-7525-5-63. 18042300 10.1186/1477-7525-5-63 PMC2222612

[84] The Children’s Society. The Good Childhood Report. 2024. https://www.childrenssociety.org.uk/information/professionals/resources/good-childhood-report-2024.

[85] Thomson A, Harriss E, Peters-Corbett A, Koppel K, Creswell C. Barriers and facilitators of community-based implementation of evidence-based interventions in the UK, for children and young people’s mental health promotion, prevention and treatment: rapid scoping review. BJPsych Open. 2023. 10.1192/bjo.2023.531. 37485912 10.1192/bjo.2023.531 PMC10375901

[86] TRL (formerly Transport Research Laboratory). Modelling the impacts of bikeability cycle training delivery on KSIs. 2024. https://www.roadsafetyknowledgecentre.org.uk/modelling-the-impacts-of-bikeability-training-on-ksis/.

[87] UK Parliament. Horizon Scanning: Active Travel. Retrieved from 2025. https://post.parliament.uk/active-travel/. 23rd June 2025.

[88] UK Parliament. The Water Safety (Curriculum) Bill. Retrieved from 2023. https://bills.parliament.uk/bills/3185. 25th June 2025.

[89] Vizard P, Obolenskaya P, Burchardt T. Child poverty amongst young carers in the UK: prevalence and trends in the wake of the financial crisis, economic downturn and onset of austerity. Child Indic Res. 2019;12:1831–54. 10.1007/s12187-018-9608-6.

[90] Welk GJ, Kim Y, Stanfill B, Osthus DA, Calabro MA, Nusser SM, Carriquiry A. Validity of 24-h physical activity recall: physical activity measurement survey. Med Sci Sports Exerc. 2014;46(10):2014–24. 10.1249/MSS.0000000000000314. 24561818 10.1249/MSS.0000000000000314 PMC4138303

[91] World Health Organization. Promoting well-being. 2025. Retrieved from https://www.who.int/activities/promoting-well-being.

