# Supplementary material for: Bikeability cycle training: a route to increasing young people’s subjective wellbeing? A retrospective cohort study
Source: BMC Public Health. 2025 Aug 18;25:2826. doi: 10.1186/s12889-025-23838-2 (PMC12359844; doi:10.1186/s12889-025-23838-2)
Supplement: Supplementary file 3 — Supplementary Material 3. [file 12889_2025_23838_MOESM3_ESM.pdf]

...	gender	PARENT_CYCLE_TRAINING_...	BIKEABILITY_L2_CODE	Can_Cycle	Cycle_Access	POSITIVE_ATTITUDE_CHILD	POSITIVE_ATTITUDE_PARENT	NEGATIVE_EXPERIENCES
1	Female	0	0	Yes	Yes	11	12	1.00
2	Female	0	0	No	No	13	7	3.29
3	Male	1	0	Yes	Yes	26	18	1.00
4	Female	0	0	Yes	Yes	21	23	1.40
5	Female	1	0	Yes	Yes	21	19	1.33
6	Female	0	0	Yes	No	15	24	.00
7	Female	0	0	Yes	Yes	22	21	.00
8	Male	0	0	Yes	No	21	8	2.00
9	Male	0	0	No	Yes	20	15	2.00
10	Male	1	0	Yes	No	26	26	.00
11	Female	1	0	Yes	No	29	29	1.00
12	Male	1	1	Yes	Yes	18	18	2.00
13	Female	0	1	Yes	Yes	16	14	1.17
14	Female	1	0	Yes	Yes	10	20	.57
15	Male	0	0	Yes	Yes	29	22	1.67
16	Female	1	1	Yes	Yes	21	23	1.50
17	Female	1	0	Yes	Yes	9	16	3.20
18	Female	1	0	No	No	15	10	.22
19	Female	1	1	Yes	Yes	15	13	1.30
20	Male	1	1	Yes	Yes	30	30	.40
21	Female	1	1	Yes	Yes	14	12	2.00
22	Male	0	0	No	No	14	14	1.00
23	Female	0	1	Yes	No	26	21	.00
24	Male	1	1	Yes	Yes	19	24	1.67
25	Female	1	1	Yes	Yes	15	22	1.10
26	Female	0	1	Yes	Yes	19	20	1.00
27	Female	0	0	Yes	No	25	15	1.00
28	Female	0	0	Yes	Yes	18	17	.00
29	Male	1	1	Yes	Yes	24	24	.00
30	Male	0	1	Yes	Yes	21	24	3.60
31	Female	0	1	Yes	Yes	12	17	1.50
32	Female	1	1	Yes	Yes	20	22	1.17
33	Female	1	1	Yes	Yes	17	16	.86
34	Male	1	1	Yes	Yes	22	20	.30
35	Female	0	0	No	No	16	16	.20
36	Male	0	1	Yes	Yes	18	14	2.00
37	Female	0	1	Yes	Yes	11	8	2.33
38	Male	0	0	Yes	No	18	19	2.00
39	Female	1	0	Yes	Yes	23	28	1.50
40	Female	0	1	Yes	Yes	17	10	2.20
41	Female	1	1	Yes	No	9	13	3.00

	MVPA	SEDENTARY	LIFE_SATISFACTION	WELLBEING	SATISFACTION_NOW	SATISFACTION_TEN_YEARS	Life_Satisfaction_Single_Item	Child_Cycling_Frequency
1	0	40	1	29	35	36	.00	4.00
2	4	5	8	34	17	16	5.00	1.00
3	4	75	48	55	39	39	9.00	5.00
4	7	14	43	63	13	22	8.00	5.00
5	42	76	27	58	21	25	7.00	3.00
6	8	70	26	45	16	19	5.00	1.00
7	3	50	3	35	26	32	1.00	2.00
8	4	39	29	48	25	32	7.00	1.00
9	15	65	7	34	27	27	1.00	3.00
10	31	55	54	50	28	27	9.00	6.00
11	15	11	37	59	21	22	8.00	5.00
12	29	45	4	48	24	30	3.00	5.00
13	40	54	27	46	22	29	4.00	3.00
14	6	32	18	46	27	28	3.00	3.00
15	42	47	44	58	31	31	9.00	4.00
16	45	57	38	50	32	32	7.00	5.00
17	15	62	27	34	18	23	5.00	5.00
18	23	48	32	46	12	12	8.00	1.00
19	23	36	46	64	13	13	8.00	3.00
20	19	26	47	62	34	35	10.00	6.00
21	39	27	37	61	27	23	8.00	3.00
22	31	27	21	49	27	36	6.00	1.00
23	10	8	30	53	18	23	5.00	1.00
24	43	38	50	64	28	31	9.00	4.00
25	13	58	19	48	33	35	3.00	1.00
26	48	40	50	61	29	30	10.00	4.00
27	19	40	28	60	25	23	7.00	2.00
28	41	18	49	70	33	32	10.00	1.00
29	14	12	46	55	16	21	8.00	5.00
30	23	24	29	44	23	19	7.00	1.00
31	7	67	24	45	25	29	8.00	3.00
32	10	37	39	69	31	34	10.00	5.00
33	18	39	6	39	24	20	4.00	5.00
34	24	7	40	58	38	32	9.00	5.00
35	10	15	22	50	26	36	5.00	1.00
36	29	36	55	68	24	33	9.00	6.00
37	17	14	48	62	21	21	8.00	1.00
38	19	42	24	47	27	30	2.00	2.00
39	16	15	58	70	35	37	10.00	5.00
40	20	27	41	68	22	24	10.00	4.00
41	4	52	30	41	21	24	8.00	1.00

	Child_Walking_Frequency	Parent_Cycling_Frequency	Parent_Walking_Frequency	ZPARENT_CYCLE_TRAINING_CODE
1	5.00	1.00	6.00	-.82281
2	3.00	1.00	1.00	-.82281
3	6.00	3.00	5.00	1.20943
4	6.00	5.00	6.00	-.82281
5	6.00	1.00	6.00	1.20943
6	6.00	1.00	6.00	-.82281
7	6.00	2.00	6.00	-.82281
8	6.00	1.00	6.00	-.82281
9	4.00	2.00	4.00	-.82281
10	6.00	5.00	6.00	1.20943
11	6.00	5.00	6.00	1.20943
12	6.00	1.00	1.00	1.20943
13	6.00	2.00	6.00	-.82281
14	4.00	2.00	5.00	1.20943
15	6.00	4.00	6.00	-.82281
16	6.00	3.00	6.00	1.20943
17	5.00	1.00	5.00	1.20943
18	6.00	1.00	6.00	1.20943
19	6.00	2.00	6.00	1.20943
20	6.00	5.00	6.00	1.20943
21	5.00	1.00	5.00	1.20943
22	1.00	1.00	1.00	-.82281
23	6.00	1.00	6.00	-.82281
24	6.00	4.00	6.00	1.20943
25	6.00	1.00	4.00	1.20943
26	6.00	5.00	6.00	-.82281
27	6.00	1.00	6.00	-.82281
28	6.00	1.00	6.00	-.82281
29	5.00	1.00	6.00	1.20943
30	1.00	1.00	1.00	-.82281
31	5.00	1.00	4.00	-.82281
32	5.00	3.00	6.00	1.20943
33	6.00	5.00	6.00	1.20943
34	6.00	4.00	6.00	1.20943
35	6.00	1.00	6.00	-.82281
36	6.00	3.00	5.00	-.82281
37	5.00	1.00	4.00	-.82281
38	6.00	2.00	6.00	-.82281
39	6.00	5.00	6.00	1.20943
40	6.00	1.00	6.00	-.82281
41	4.00	1.00	6.00	1.20943

	ZBIKEABILITY_L2_CODE	ZPOSITIVE_ATTITUDE_CHILD	ZPOSITIVE_ATTITUDE_PARENT
1	-1.12763	-1.96428	-1.27680
2	-1.12763	-1.43022	-2.47307
3	-1.12763	2.04113	.15872
4	-1.12763	.70599	1.35500
5	-1.12763	.70599	.39798
6	-1.12763	-.89617	1.59425
7	-1.12763	.97302	.87649
8	-1.12763	.70599	-2.23382
9	-1.12763	.43897	-.55904
10	-1.12763	2.04113	2.07276
11	-1.12763	2.84221	2.79052
12	.88249	-.09509	.15872
13	.88249	-.62914	-.79829
14	-1.12763	-2.23131	.63723
15	-1.12763	2.84221	1.11574
16	.88249	.70599	1.35500
17	-1.12763	-2.49833	-.31978
18	-1.12763	-.89617	-1.75531
19	.88249	-.89617	-1.03755
20	.88249	3.10924	3.02978
21	.88249	-1.16320	-1.27680
22	-1.12763	-1.16320	-.79829
23	.88249	2.04113	.87649
24	.88249	.17194	1.59425
25	.88249	-.89617	1.11574
26	.88249	.17194	.63723
27	-1.12763	1.77410	-.55904
28	-1.12763	-.09509	-.08053
29	.88249	1.50708	1.59425
30	.88249	.70599	1.59425
31	.88249	-1.69725	-.08053
32	.88249	.43897	1.11574
33	.88249	-.36212	-.31978
34	.88249	.97302	.63723
35	-1.12763	-.62914	-.31978
36	.88249	-.09509	-.79829
37	.88249	-1.96428	-2.23382
38	-1.12763	-.09509	.39798
39	-1.12763	1.24005	2.55127
40	.88249	-.36212	-1.75531
41	.88249	-2.49833	-1.03755

	ZNEGATIVE_EXPERIENCES	ZMVPA	ZSEDENTARY	ZLIFE_SATISFACTION
1	-.16159	-1.76583	.25595	-3.11237
2	2.77121	-1.33035	-1.82622	-2.53941
3	-.16159	-1.33035	2.33813	.73467
4	.35165	-1.00373	-1.29080	.32541
5	.26611	2.80674	2.39762	-.98422
6	-1.44470	-.89486	2.04067	-1.06607
7	-1.44470	-1.43922	.85086	-2.94867
8	1.12151	-1.33035	.19646	-.82052
9	1.12151	-.13277	1.74322	-2.62126
10	-1.44470	1.60916	1.14831	1.22578
11	-.16159	-.13277	-1.46927	-.16570
12	1.12151	1.39142	.55341	-2.86682
13	.05226	2.58900	1.08882	-.98422
14	-.71149	-1.11260	-.21997	-1.72089
15	.69381	2.80674	.67239	.40726
16	.47996	3.13335	1.26730	-.08385
17	2.66123	-.13277	1.56475	-.98422
18	-1.15956	.73820	.73188	-.57496
19	.22334	.73820	.01799	.57097
20	-.93146	.30271	-.57691	.65282
21	1.12151	2.48012	-.51742	-.16570
22	-.16159	1.60916	-.51742	-1.47533
23	-1.44470	-.67712	-1.64774	-.73866
24	.69381	2.91561	.13697	.89838
25	-.03328	-.35051	1.32679	-1.63904
26	-.16159	3.45996	.25595	.89838
27	-.16159	.30271	.25595	-.90237
28	-1.44470	2.69787	-1.05284	.81652
29	-1.44470	-.24164	-1.40978	.57097
30	3.17447	.73820	-.69589	-.82052
31	.47996	-1.00373	1.86220	-1.22978
32	.05226	-.67712	.07748	-.00200
33	-.34489	.19384	.19646	-2.70311
34	-1.05977	.84707	-1.70724	.07986
35	-1.18808	-.67712	-1.23131	-1.39348
36	1.12151	1.39142	.01799	1.30764
37	1.54921	.08497	-1.29080	.73467
38	1.12151	.30271	.37494	-1.22978
39	.47996	-.02390	-1.23131	1.55319
40	1.37813	.41158	-.51742	.16171
41	2.40461	-1.33035	.96984	-.73866

	ZWELLBEING	ZSATISFACTION_NOW	ZSATISFACTION_TEN_YEARS
1	-2.94348	1.47418	1.49429
2	-2.36902	-1.89616	-2.34924
3	.04372	2.22315	2.07082
4	.96285	-2.64513	-1.19618
5	.38839	-1.14720	-.61965
6	-1.10521	-2.08340	-1.77271
7	-2.25413	-.21099	.72558
8	-.76053	-.39823	.72558
9	-2.36902	-.02375	-.23530
10	-.53075	.16349	-.23530
11	.50328	-1.14720	-1.19618
12	-.76053	-.58547	.34123
13	-.99031	-.95995	.14905
14	-.99031	-.02375	-.04312
15	.38839	.72522	.53341
16	-.53075	.91246	.72558
17	-2.36902	-1.70892	-1.00401
18	-.99031	-2.83237	-3.11795
19	1.07774	-2.64513	-2.92577
20	.84796	1.28694	1.30211
21	.73307	-.02375	-1.00401
22	-.64564	-.02375	1.49429
23	-.18607	-1.70892	-1.00401
24	1.07774	.16349	.53341
25	-.76053	1.09970	1.30211
26	.73307	.35074	.34123
27	.61818	-.39823	-1.00401
28	1.76710	1.09970	.72558
29	.04372	-2.08340	-1.38836
30	-1.22010	-.77271	-1.77271
31	-1.10521	-.39823	.14905
32	1.65220	.72522	1.10994
33	-1.79456	-.58547	-1.58054
34	.38839	2.03591	.72558
35	-.53075	-.21099	1.49429
36	1.53731	-.58547	.91776
37	.84796	-1.14720	-1.38836
38	-.87542	-.02375	.34123
39	1.76710	1.47418	1.68647
40	1.53731	-.95995	-.81183
41	-1.56477	-1.14720	-.81183

	ZLife_Satisfaction_Single_Item	ZChild_Cycling_Frequency
1	-3.74252	.32118
2	-1.29448	-1.44967
3	.66396	.91147
4	.17435	.91147
5	-.31526	-.26910
6	-1.29448	-1.44967
7	-3.25291	-.85939
8	-.31526	-1.44967
9	-3.25291	-.26910
10	.66396	1.50175
11	.17435	.91147
12	-2.27369	.91147
13	-1.78409	-.26910
14	-2.27369	-.26910
15	.66396	.32118
16	-.31526	.91147
17	-1.29448	.91147
18	.17435	-1.44967
19	.17435	-.26910
20	1.15357	1.50175
21	.17435	-.26910
22	-.80487	-1.44967
23	-1.29448	-1.44967
24	.66396	.32118
25	-2.27369	-1.44967
26	1.15357	.32118
27	-.31526	-.85939
28	1.15357	-1.44967
29	.17435	.91147
30	-.31526	-1.44967
31	.17435	-.26910
32	1.15357	.91147
33	-1.78409	.91147
34	.66396	.91147
35	-1.29448	-1.44967
36	.66396	1.50175
37	.17435	-1.44967
38	-2.76330	-.85939
39	1.15357	.91147
40	1.15357	.32118
41	.17435	-1.44967

	ZChild_Walking_Frequency	ZParent_Cycling_Frequency
1	-.61013	-.88951
2	-2.79376	-.88951
3	.48168	.47328
4	.48168	1.83607
5	.48168	-.88951
6	.48168	-.88951
7	.48168	-.20811
8	.48168	-.88951
9	-1.70194	-.20811
10	.48168	1.83607
11	.48168	1.83607
12	.48168	-.88951
13	.48168	-.20811
14	-1.70194	-.20811
15	.48168	1.15468
16	.48168	.47328
17	-.61013	-.88951
18	.48168	-.88951
19	.48168	-.20811
20	.48168	1.83607
21	-.61013	-.88951
22	-4.97738	-.88951
23	.48168	-.88951
24	.48168	1.15468
25	.48168	-.88951
26	.48168	1.83607
27	.48168	-.88951
28	.48168	-.88951
29	-.61013	-.88951
30	-4.97738	-.88951
31	-.61013	-.88951
32	-.61013	.47328
33	.48168	1.83607
34	.48168	1.15468
35	.48168	-.88951
36	.48168	.47328
37	-.61013	-.88951
38	.48168	-.20811
39	.48168	1.83607
40	.48168	-.88951
41	-1.70194	-.88951

	ZParent_Walking_Frequency	MAH_2	Probability_MAH_2	filter_$
1	.52943	23.46829	.0052	Selected
2	-4.42490	22.51268	.0074	Selected
3	-.46143	21.92023	.0091	Selected
4	.52943	18.57999	.0290	Selected
5	.52943	18.41732	.0306	Selected
6	.52943	17.69769	.0388	Selected
7	.52943	17.29930	.0442	Selected
8	.52943	17.03456	.0482	Selected
9	-1.45230	16.98389	.0490	Selected
10	.52943	16.77445	.0524	Selected
11	.52943	16.47413	.0576	Selected
12	-4.42490	16.45624	.0579	Selected
13	.52943	15.86866	.0697	Selected
14	-.46143	15.53051	.0774	Selected
15	.52943	15.35602	.0816	Selected
16	.52943	15.21348	.0852	Selected
17	-.46143	15.19556	.0857	Selected
18	.52943	14.79443	.0967	Selected
19	.52943	14.73392	.0985	Selected
20	.52943	14.61201	.1022	Selected
21	-.46143	14.35977	.1101	Selected
22	-4.42490	14.35904	.1101	Selected
23	.52943	14.17490	.1162	Selected
24	.52943	13.57863	.1381	Selected
25	-1.45230	13.56849	.1385	Selected
26	.52943	13.42036	.1445	Selected
27	.52943	12.99370	.1629	Selected
28	.52943	12.60774	.1812	Selected
29	.52943	12.55125	.1840	Selected
30	-4.42490	12.34817	.1944	Selected
31	-1.45230	12.28316	.1978	Selected
32	.52943	12.24073	.2001	Selected
33	.52943	12.21927	.2012	Selected
34	.52943	12.13193	.2060	Selected
35	.52943	12.12503	.2064	Selected
36	-.46143	12.05509	.2102	Selected
37	-1.45230	11.91663	.2181	Selected
38	.52943	11.90439	.2188	Selected
39	.52943	11.85650	.2215	Selected
40	.52943	11.77347	.2264	Selected
41	.52943	11.45626	.2457	Selected

	ZSco01	ZSco02	ZSco03	ZSco04	ZSco05
1	-.83659	-1.13035	-1.94795	-1.26488	-.15141
2	-.83659	-1.13035	-1.41813	-2.45514	2.77098
3	1.18938	-1.13035	2.02571	.16344	-.15141
4	-.83659	-1.13035	.70116	1.35370	.36001
5	1.18938	-1.13035	.70116	.40149	.27477
6	-.83659	-1.13035	-.88831	1.59176	-1.42995
7	-.83659	-1.13035	.96607	.87760	-1.42995
8	-.83659	-1.13035	.70116	-2.21709	1.12714
9	-.83659	-1.13035	.43625	-.55072	1.12714
10	1.18938	-1.13035	2.02571	2.06786	-1.42995
11	1.18938	-1.13035	2.82044	2.78202	-.15141
12	1.18938	.88028	-.09358	.16344	1.12714
13	-.83659	.88028	-.62340	-.78877	.06168
14	1.18938	-1.13035	-2.21286	.63955	-.69936
15	-.83659	-1.13035	2.82044	1.11565	.70096
16	1.18938	.88028	.70116	1.35370	.48787
17	1.18938	-1.13035	-2.47777	-.31267	2.66139
18	1.18938	-1.13035	-.88831	-1.74098	-1.14583
19	1.18938	.88028	-.88831	-1.02683	.23216
20	1.18938	.88028	3.08535	3.02007	-.91853
21	1.18938	.88028	-1.15322	-1.26488	1.12714
22	-.83659	-1.13035	-1.15322	-.78877	-.15141
23	-.83659	.88028	2.02571	.87760	-1.42995
24	1.18938	.88028	.17133	1.59176	.70096
25	1.18938	.88028	-.88831	1.11565	-.02355
26	-.83659	.88028	.17133	.63955	-.15141
27	-.83659	-1.13035	1.76080	-.55072	-.15141
28	-.83659	-1.13035	-.09358	-.07461	-1.42995
29	1.18938	.88028	1.49589	1.59176	-1.42995
30	-.83659	.88028	.70116	1.59176	3.17281
31	-.83659	.88028	-1.68304	-.07461	.48787
32	1.18938	.88028	.43625	1.11565	.06168
33	1.18938	.88028	-.35849	-.31267	-.33406
34	1.18938	.88028	.96607	.63955	-1.04639
35	-.83659	-1.13035	-.62340	-.31267	-1.17424
36	-.83659	.88028	-.09358	-.78877	1.12714
37	-.83659	.88028	-1.94795	-2.21709	1.55332
38	-.83659	-1.13035	-.09358	.40149	1.12714
39	1.18938	-1.13035	1.23098	2.54397	.48787
40	-.83659	.88028	-.35849	-1.74098	1.38285
41	1.18938	.88028	?	?	?

	ZSco06	ZSco07	ZSco08	ZSco09	ZSco10
1	-1.75556	.24635	-3.17212	-2.92953	1.48851
2	-1.32259	-1.83665	-2.59196	-2.35584	-1.92431
3	-1.32259	2.32935	.72324	.05366	2.24691
4	-.99787	-1.30102	.30884	.97156	-2.68271
5	2.79058	2.38886	-1.01724	.39787	-1.16590
6	-.88963	2.03178	-1.10012	-1.09372	-2.11391
7	-1.43083	.84149	-3.00636	-2.24110	-.21790
8	-1.32259	.18683	-.85148	-.74951	-.40750
9	-.13194	1.73420	-2.67484	-2.35584	-.02830
10	1.59993	1.13906	1.22052	-.52003	.16130
11	-.13194	-1.47957	-.18844	.51261	-1.16590
12	1.38344	.54392	-2.92348	-.74951	-.59710
13	2.57410	1.07955	-1.01724	-.97898	-.97630
14	-1.10611	-.22977	-1.76316	-.97898	-.02830
15	2.79058	.66295	.39172	.39787	.73010
16	3.11531	1.25809	-.10556	-.52003	.91971
17	-.13194	1.55566	-1.01724	-2.35584	-1.73471
18	.73400	.72246	-.60284	-.97898	-2.87231
19	.73400	.00829	.55748	1.08630	-2.68271
20	.30103	-.58685	.64036	.85682	1.29891
21	2.46586	-.52734	-.18844	.74209	-.02830
22	1.59993	-.52734	-1.51452	-.63477	-.02830
23	-.67314	-1.65811	-.76860	-.17582	-1.73471
24	2.89882	.12732	.88900	1.08630	.16130
25	-.34842	1.31760	-1.68028	-.74951	1.10931
26	3.44003	.24635	.88900	.74209	.35090
27	.30103	.24635	-.93436	.62735	-.40750
28	2.68234	-1.06297	.80612	1.77473	1.10931
29	-.24018	-1.42005	.55748	.05366	-2.11391
30	.73400	-.70588	-.85148	-1.20846	-.78670
31	-.99787	1.85323	-1.26588	-1.09372	-.40750
32	-.67314	.06780	-.02268	1.65999	.73010
33	.19279	.18683	-2.75772	-1.78215	-.59710
34	.84224	-1.71762	.06020	.39787	2.05731
35	-.67314	-1.24151	-1.43164	-.52003	-.21790
36	1.38344	.00829	1.30340	1.54525	-.59710
37	.08455	-1.30102	.72324	.85682	-1.16590
38	.30103	.36538	-1.26588	-.86424	-.02830
39	-.02369	-1.24151	1.55204	1.77473	1.48851
40	.40927	-.52734	.14308	1.54525	-.97630
41	-1.32259	.96052	-.76860	-1.55267	-1.16590

	ZSco11	ZSco12	ZSco13	ZSco14	ZSco15
1	1.54838	-3.79838	.31674	-.60155	-.89231
2	-2.45966	-1.31634	-1.45936	-2.76928	-.89231
3	2.14959	.66929	.90877	.48232	.46318
4	-1.25725	.17288	.90877	.48232	1.81867
5	-.65604	-.32353	-.27530	.48232	-.89231
6	-1.85846	-1.31634	-1.45936	.48232	-.89231
7	.74677	-3.30197	-.86733	.48232	-.21456
8	.74677	-.32353	-1.45936	.48232	-.89231
9	-.25524	-3.30197	-.27530	-1.68541	-.21456
10	-.25524	.66929	1.50080	.48232	1.81867
11	-1.25725	.17288	.90877	.48232	1.81867
12	.34597	-2.30916	.90877	.48232	-.89231
13	.14557	-1.81275	-.27530	.48232	-.21456
14	-.05484	-2.30916	-.27530	-1.68541	-.21456
15	.54637	.66929	.31674	.48232	1.14093
16	.74677	-.32353	.90877	.48232	.46318
17	-1.05685	-1.31634	.90877	-.60155	-.89231
18	-3.26127	.17288	-1.45936	.48232	-.89231
19	-3.06087	.17288	-.27530	.48232	-.21456
20	1.34798	1.16569	1.50080	.48232	1.81867
21	-1.05685	.17288	-.27530	-.60155	-.89231
22	1.54838	-.81994	-1.45936	-4.93702	-.89231
23	-1.05685	-1.31634	-1.45936	.48232	-.89231
24	.54637	.66929	.31674	.48232	1.14093
25	1.34798	-2.30916	-1.45936	.48232	-.89231
26	.34597	1.16569	.31674	.48232	1.81867
27	-1.05685	-.32353	-.86733	.48232	-.89231
28	.74677	1.16569	-1.45936	.48232	-.89231
29	-1.45765	.17288	.90877	-.60155	-.89231
30	-1.85846	-.32353	-1.45936	-4.93702	-.89231
31	.14557	.17288	-.27530	-.60155	-.89231
32	1.14758	1.16569	.90877	-.60155	.46318
33	-1.65805	-1.81275	.90877	.48232	1.81867
34	.74677	.66929	.90877	.48232	1.14093
35	1.54838	-1.31634	-1.45936	.48232	-.89231
36	.94717	.66929	1.50080	.48232	.46318
37	-1.45765	.17288	-1.45936	-.60155	-.89231
38	.34597	-2.80557	-.86733	.48232	-.21456
39	1.74878	1.16569	.90877	.48232	1.81867
40	-.85645	1.16569	.31674	.48232	-.89231
41	-.85645	.17288	-1.45936	-1.68541	-.89231

	ZSco16
1	.52614
2	-4.39109
3	-.45730
4	.52614
5	.52614
6	.52614
7	.52614
8	.52614
9	-1.44075
10	.52614
11	.52614
12	-4.39109
13	.52614
14	-.45730
15	.52614
16	.52614
17	-.45730
18	.52614
19	.52614
20	.52614
21	-.45730
22	-4.39109
23	.52614
24	.52614
25	-1.44075
26	.52614
27	.52614
28	.52614
29	.52614
30	-4.39109
31	-1.44075
32	.52614
33	.52614
34	.52614
35	.52614
36	-.45730
37	-1.44075
38	.52614
39	.52614
40	.52614
41	.52614

	gender	PARENT_CYCLE_TRAINING_...	BIKEABILITY_L2_CODE	Can_Cycle	Cycle_Access	POSITIVE_ATTITUDE_CHILD	POSITIVE_ATTITUDE_PARENT	NEGATIVE_EXPERIENCES
42	Male	0	0	Yes	Yes	21	19	.20
43	Female	0	1	Yes	Yes	16	13	.80
44	Male	0	1	Yes	Yes	12	15	.60
45	Female	0	0	Yes	Yes	19	17	.50
46	Female	0	0	No	No	15	19	.00
47	Female	1	0	Yes	Yes	18	17	.50
48	Female	1	1	Yes	Yes	23	19	1.20
49	Female	1	0	Yes	No	22	22	.75
50	Non-bina	0	1	Yes	Yes	13	20	3.50
51	Female	1	0	Yes	No	22	22	.71
52	Female	0	1	Yes	Yes	17	15	1.33
53	Male	0	1	Yes	Yes	23	24	.20
54	Female	0	0	Yes	Yes	18	20	1.20
55	Female	1	0	Yes	Yes	16	18	1.00
56	Male	0	0	Yes	No	21	18	.00
57	Male	1	1	Yes	Yes	13	14	2.17
58	Male	1	1	Yes	Yes	15	9	.25
59	Male	0	0	Yes	Yes	19	17	1.50
60	Female	0	0	No	No	18	18	1.00
61	Male	1	1	Yes	Yes	22	16	1.67
62	Male	1	1	Yes	Yes	23	18	1.67
63	Male	1	1	Yes	Yes	16	12	1.25
64	Female	1	1	Yes	No	14	17	3.00
65	Female	1	1	Yes	Yes	22	18	2.40
66	Female	1	1	Yes	Yes	16	18	1.00
67	Male	0	1	Yes	Yes	21	24	.86
68	Female	1	1	Yes	Yes	15	9	3.00
69	Female	0	0	No	No	18	18	.
70	Female	0	0	Yes	Yes	15	16	.43
71	Male	0	1	Yes	No	17	10	.67
72	Female	0	0	Yes	Yes	18	20	2.00
73	Female	0	1	Yes	Yes	13	12	.00
74	Male	0	0	Yes	No	19	13	1.40
75	Male	0	0	Yes	No	24	18	.00
76	Male	1	1	Yes	Yes	16	16	1.00
77	Male	1	1	Yes	Yes	23	20	.00
78	Female	0	0	Yes	No	14	13	2.00
79	Female	0	0	No	No	16	16	.50
80	Male	1	0	Yes	Yes	20	25	1.00
81	Male	0	1	Yes	No	17	14	.50
82	Female	1	1	Yes	Yes	13	10	2.67

	MVPA	SEDENTARY	LIFE_SATISFACTION	WELLBEING	SATISFACTION_NOW	SATISFACTION_TEN_YEARS	Life_Satisfaction_Single_Item	Child_Cycling_Frequency
42	13	65	49	60	24	32	8.00	4.00
43	13	65	43	57	16	22	9.00	4.00
44	6	70	29	41	34	33	6.00	4.00
45	20	37	35	67	39	38	8.00	5.00
46	13	18	50	61	18	16	10.00	1.00
47	38	53	45	54	29	35	10.00	4.00
48	12	65	43	54	37	32	8.00	6.00
49	11	13	36	54	29	29	10.00	1.00
50	10	45	49	56	37	36	9.00	4.00
51	6	44	47	51	21	28	9.00	1.00
52	13	5	42	43	23	23	8.00	1.00
53	16	23	46	51	32	29	6.00	6.00
54	12	11	41	66	34	34	6.00	6.00
55	8	69	40	58	21	21	9.00	3.00
56	11	7	60	71	39	38	10.00	1.00
57	20	61	45	49	19	27	8.00	4.00
58	19	30	49	60	36	35	8.00	5.00
59	22	14	46	61	40	38	10.00	4.00
60	35	53	31	52	24	23	9.00	1.00
61	18	21	36	43	28	30	5.00	6.00
62	11	65	30	54	23	26	8.00	5.00
63	12	16	49	51	25	22	7.00	4.00
64	4	56	19	40	25	23	3.00	3.00
65	11	62	43	49	27	28	10.00	6.00
66	7	36	54	60	20	19	8.00	4.00
67	28	21	50	52	31	31	9.00	5.00
68	17	38	16	40	25	32	4.00	2.00
69	28	21	33	47	23	24	9.00	1.00
70	9	45	36	62	26	26	10.00	6.00
71	17	42	48	57	33	32	7.00	1.00
72	15	52	49	56	31	23	9.00	4.00
73	23	13	47	52	30	28	9.00	3.00
74	12	52	28	43	23	30	8.00	2.00
75	34	36	30	54	24	25	8.00	2.00
76	31	62	36	55	21	23	6.00	4.00
77	36	12	57	68	28	28	9.00	5.00
78	1	31	40	53	21	23	10.00	1.00
79	7	13	11	38	22	24	3.00	1.00
80	8	31	21	42	23	26	4.00	5.00
81	6	30	21	43	31	34	7.00	1.00
82	11	22	43	53	24	30	6.00	1.00

	Child_Walking_Frequency	Parent_Cycling_Frequency	Parent_Walking_Frequency	ZPARENT_CYCLE_TRAINING_CODE
42	6.00	3.00	6.00	-.82281
43	6.00	1.00	6.00	-.82281
44	6.00	1.00	6.00	-.82281
45	6.00	2.00	6.00	-.82281
46	5.00	1.00	5.00	-.82281
47	6.00	1.00	6.00	1.20943
48	6.00	5.00	5.00	1.20943
49	5.00	1.00	6.00	1.20943
50	6.00	1.00	6.00	-.82281
51	4.00	1.00	4.00	1.20943
52	1.00	2.00	1.00	-.82281
53	6.00	1.00	6.00	-.82281
54	6.00	1.00	6.00	-.82281
55	4.00	3.00	4.00	1.20943
56	1.00	1.00	5.00	-.82281
57	5.00	5.00	6.00	1.20943
58	6.00	1.00	6.00	1.20943
59	5.00	2.00	6.00	-.82281
60	6.00	1.00	6.00	-.82281
61	6.00	4.00	6.00	1.20943
62	6.00	5.00	6.00	1.20943
63	5.00	1.00	6.00	1.20943
64	6.00	1.00	6.00	1.20943
65	6.00	6.00	6.00	1.20943
66	6.00	3.00	5.00	1.20943
67	6.00	5.00	6.00	-.82281
68	6.00	1.00	6.00	1.20943
69	6.00	2.00	6.00	-.82281
70	6.00	1.00	6.00	-.82281
71	6.00	1.00	5.00	-.82281
72	4.00	1.00	6.00	-.82281
73	5.00	1.00	6.00	-.82281
74	5.00	1.00	6.00	-.82281
75	6.00	1.00	6.00	-.82281
76	6.00	4.00	6.00	1.20943
77	6.00	3.00	6.00	1.20943
78	5.00	1.00	6.00	-.82281
79	6.00	.	5.00	-.82281
80	6.00	3.00	4.00	1.20943
81	6.00	1.00	5.00	-.82281
82	6.00	1.00	5.00	1.20943

	ZBIKEABILITY_L2_CODE	ZPOSITIVE_ATTITUDE_CHILD	ZPOSITIVE_ATTITUDE_PARENT
42	-1.12763	.70599	.39798
43	.88249	-.62914	-1.03755
44	.88249	-1.69725	-.55904
45	-1.12763	.17194	-.08053
46	-1.12763	-.89617	.39798
47	-1.12763	-.09509	-.08053
48	.88249	1.24005	.39798
49	-1.12763	.97302	1.11574
50	.88249	-1.43022	.63723
51	-1.12763	.97302	1.11574
52	.88249	-.36212	-.55904
53	.88249	1.24005	1.59425
54	-1.12763	-.09509	.63723
55	-1.12763	-.62914	.15872
56	-1.12763	.70599	.15872
57	.88249	-1.43022	-.79829
58	.88249	-.89617	-1.99456
59	-1.12763	.17194	-.08053
60	-1.12763	-.09509	.15872
61	.88249	.97302	-.31978
62	.88249	1.24005	.15872
63	.88249	-.62914	-1.27680
64	.88249	-1.16320	-.08053
65	.88249	.97302	.15872
66	.88249	-.62914	.15872
67	.88249	.70599	1.59425
68	.88249	-.89617	-1.99456
69	-1.12763	-.09509	.15872
70	-1.12763	-.89617	-.31978
71	.88249	-.36212	-1.75531
72	-1.12763	-.09509	.63723
73	.88249	-1.43022	-1.27680
74	-1.12763	.17194	-1.03755
75	-1.12763	1.50708	.15872
76	.88249	-.62914	-.31978
77	.88249	1.24005	.63723
78	-1.12763	-1.16320	-1.03755
79	-1.12763	-.62914	-.31978
80	-1.12763	.43897	1.83351
81	.88249	-.36212	-.79829
82	.88249	-1.43022	-1.75531

	ZNEGATIVE_EXPERIENCES	ZMVPA	ZSEDENTARY	ZLIFE_SATISFACTION
42	-1.18808	-.35051	1.74322	.81652
43	-.41821	-.35051	1.74322	.32541
44	-.67483	-1.11260	2.04067	-.82052
45	-.80315	.41158	.07748	-.32940
46	-1.44470	-.35051	-1.05284	.89838
47	-.80315	2.37125	1.02933	.48912
48	.09503	-.45938	1.74322	.32541
49	-.48237	-.56825	-1.35029	-.24755
50	3.04616	-.67712	.55341	.81652
51	-.52819	-1.11260	.49392	.65282
52	.26611	-.35051	-1.82622	.24356
53	-1.18808	-.02390	-.75539	.57097
54	.09503	-.45938	-1.46927	.16171
55	-.16159	-.89486	1.98118	.07986
56	-1.44470	-.56825	-1.70724	1.71690
57	1.33536	.41158	1.50526	.48912
58	-1.12392	.30271	-.33895	.81652
59	.47996	.62932	-1.29080	.57097
60	-.16159	2.04464	1.02933	-.65681
61	.69381	.19384	-.87437	-.24755
62	.69381	-.56825	1.74322	-.73866
63	.15918	-.45938	-1.17182	.81652
64	2.40461	-1.33035	1.20780	-1.63904
65	1.63475	-.56825	1.56475	.32541
66	-.16159	-1.00373	.01799	1.22578
67	-.34489	1.28255	-.87437	.89838
68	2.40461	.08497	.13697	-1.88459
69	.	1.28255	-.87437	-.49311
70	-.89480	-.78599	.55341	-.24755
71	-.58929	.08497	.37494	.73467
72	1.12151	-.13277	.96984	.81652
73	-1.44470	.73820	-1.35029	.65282
74	.35165	-.45938	.96984	-.90237
75	-1.44470	1.93577	.01799	-.73866
76	-.16159	1.60916	1.56475	-.24755
77	-1.44470	2.15351	-1.40978	1.47134
78	1.12151	-1.65696	-.27946	.07986
79	-.80315	-1.00373	-1.35029	-2.29385
80	-.16159	-.89486	-.27946	-1.47533
81	-.80315	-1.11260	-.33895	-1.47533
82	1.97691	-.56825	-.81488	.32541

	ZWELLBEING	ZSATISFACTION_NOW	ZSATISFACTION_TEN_YEARS
42	.61818	-.58547	.72558
43	.27350	-2.08340	-1.19618
44	-1.56477	1.28694	.91776
45	1.42242	2.22315	1.87865
46	.73307	-1.70892	-2.34924
47	-.07118	.35074	1.30211
48	-.07118	1.84867	.72558
49	-.07118	.35074	.14905
50	.15861	1.84867	1.49429
51	-.41585	-1.14720	-.04312
52	-1.33499	-.77271	-1.00401
53	-.41585	.91246	.14905
54	1.30753	1.28694	1.10994
55	.38839	-1.14720	-1.38836
56	1.88199	2.22315	1.87865
57	-.64564	-1.52168	-.23530
58	.61818	1.66143	1.30211
59	.73307	2.41039	1.87865
60	-.30096	-.58547	-1.00401
61	-1.33499	.16349	.34123
62	-.07118	-.77271	-.42748
63	-.41585	-.39823	-1.19618
64	-1.67967	-.39823	-1.00401
65	-.64564	-.02375	-.04312
66	.61818	-1.33444	-1.77271
67	-.30096	.72522	.53341
68	-1.67967	-.39823	.72558
69	-.87542	-.77271	-.81183
70	.84796	-.21099	-.42748
71	.27350	1.09970	.72558
72	.15861	.72522	-1.00401
73	-.30096	.53798	-.04312
74	-1.33499	-.77271	.34123
75	-.07118	-.58547	-.61965
76	.04372	-1.14720	-1.00401
77	1.53731	.16349	-.04312
78	-.18607	-1.14720	-1.00401
79	-1.90945	-.95995	-.81183
80	-1.44988	-.77271	-.42748
81	-1.33499	.72522	1.10994
82	-.18607	-.58547	.34123

	ZLife_Satisfaction_Single_Item	ZChild_Cycling_Frequency
42	.17435	.32118
43	.66396	.32118
44	-.80487	.32118
45	.17435	.91147
46	1.15357	-1.44967
47	1.15357	.32118
48	.17435	1.50175
49	1.15357	-1.44967
50	.66396	.32118
51	.66396	-1.44967
52	.17435	-1.44967
53	-.80487	1.50175
54	-.80487	1.50175
55	.66396	-.26910
56	1.15357	-1.44967
57	.17435	.32118
58	.17435	.91147
59	1.15357	.32118
60	.66396	-1.44967
61	-1.29448	1.50175
62	.17435	.91147
63	-.31526	.32118
64	-2.27369	-.26910
65	1.15357	1.50175
66	.17435	.32118
67	.66396	.91147
68	-1.78409	-.85939
69	.66396	-1.44967
70	1.15357	1.50175
71	-.31526	-1.44967
72	.66396	.32118
73	.66396	-.26910
74	.17435	-.85939
75	.17435	-.85939
76	-.80487	.32118
77	.66396	.91147
78	1.15357	-1.44967
79	-2.27369	-1.44967
80	-1.78409	.91147
81	-.31526	-1.44967
82	-.80487	-1.44967

	ZChild_Walking_Frequency	ZParent_Cycling_Frequency
42	.48168	.47328
43	.48168	-.88951
44	.48168	-.88951
45	.48168	-.20811
46	-.61013	-.88951
47	.48168	-.88951
48	.48168	1.83607
49	-.61013	-.88951
50	.48168	-.88951
51	-1.70194	-.88951
52	-4.97738	-.20811
53	.48168	-.88951
54	.48168	-.88951
55	-1.70194	.47328
56	-4.97738	-.88951
57	-.61013	1.83607
58	.48168	-.88951
59	-.61013	-.20811
60	.48168	-.88951
61	.48168	1.15468
62	.48168	1.83607
63	-.61013	-.88951
64	.48168	-.88951
65	.48168	2.51747
66	.48168	.47328
67	.48168	1.83607
68	.48168	-.88951
69	.48168	-.20811
70	.48168	-.88951
71	.48168	-.88951
72	-1.70194	-.88951
73	-.61013	-.88951
74	-.61013	-.88951
75	.48168	-.88951
76	.48168	1.15468
77	.48168	.47328
78	-.61013	-.88951
79	.48168	.
80	.48168	.47328
81	.48168	-.88951
82	.48168	-.88951

	ZParent_Walking_Frequency	MAH_2	Probability_MAH_2	filter_$
42	.52943	11.39158	.2498	Selected
43	.52943	11.37157	.2511	Selected
44	.52943	11.25644	.2585	Selected
45	.52943	11.02609	.2739	Selected
46	-.46143	10.99413	.2761	Selected
47	.52943	10.96494	.2781	Selected
48	-.46143	10.86949	.2848	Selected
49	.52943	10.76900	.2919	Selected
50	.52943	10.72595	.2950	Selected
51	-1.45230	10.59255	.3047	Selected
52	-4.42490	10.49738	.3117	Selected
53	.52943	10.45172	.3152	Selected
54	.52943	10.43394	.3165	Selected
55	-1.45230	10.42822	.3169	Selected
56	-.46143	10.40589	.3186	Selected
57	.52943	10.31620	.3255	Selected
58	.52943	10.25431	.3303	Selected
59	.52943	10.24652	.3309	Selected
60	.52943	10.19724	.3348	Selected
61	.52943	10.13476	.3397	Selected
62	.52943	9.96100	.3537	Selected
63	.52943	9.82656	.3647	Selected
64	.52943	9.76526	.3698	Selected
65	.52943	9.69792	.3755	Selected
66	-.46143	9.66706	.3781	Selected
67	.52943	9.59575	.3842	Selected
68	.52943	9.53037	.3898	Selected
69	.52943	9.52378	.3904	Selected
70	.52943	9.51667	.3910	Selected
71	-.46143	9.49495	.3929	Selected
72	.52943	9.45954	.3960	Selected
73	.52943	9.39762	.4014	Selected
74	.52943	9.38020	.4029	Selected
75	.52943	9.24963	.4146	Selected
76	.52943	9.23414	.4159	Selected
77	.52943	9.23008	.4163	Selected
78	.52943	9.22535	.4167	Selected
79	-.46143	9.15695	.4229	Selected
80	-1.45230	9.12778	.4256	Selected
81	-.46143	9.02345	.4351	Selected
82	-.46143	9.00255	.4370	Selected

	ZSco01	ZSco02	ZSco03	ZSco04	ZSco05
42	-.83659	-1.13035	.70116	.40149	-1.17424
43	-.83659	.88028	-.62340	-1.02683	-.40712
44	-.83659	.88028	-1.68304	-.55072	-.66283
45	-.83659	-1.13035	.17133	-.07461	-.79068
46	-.83659	-1.13035	-.88831	.40149	-1.42995
47	1.18938	-1.13035	-.09358	-.07461	-.79068
48	1.18938	.88028	1.23098	.40149	.10430
49	1.18938	-1.13035	.96607	1.11565	-.47104
50	-.83659	.88028	-1.41813	.63955	3.04496
51	1.18938	-1.13035	.96607	1.11565	-.51671
52	-.83659	.88028	-.35849	-.55072	.27477
53	-.83659	.88028	1.23098	1.59176	-1.17424
54	-.83659	-1.13035	-.09358	.63955	.10430
55	1.18938	-1.13035	-.62340	.16344	-.15141
56	-.83659	-1.13035	.70116	.16344	-1.42995
57	1.18938	.88028	-1.41813	-.78877	1.34023
58	1.18938	.88028	-.88831	-1.97904	-1.11032
59	-.83659	-1.13035	.17133	-.07461	.48787
60	-.83659	-1.13035	-.09358	.16344	-.15141
61	1.18938	.88028	.96607	-.31267	.70096
62	1.18938	.88028	1.23098	.16344	.70096
63	1.18938	.88028	-.62340	-1.26488	.16823
64	1.18938	.88028	-1.15322	-.07461	2.40568
65	1.18938	.88028	.96607	.16344	1.63856
66	1.18938	.88028	-.62340	.16344	-.15141
67	-.83659	.88028	.70116	1.59176	-.33406
68	1.18938	.88028	-.88831	-1.97904	2.40568
69	-.83659	-1.13035	-.09358	.16344	.
70	-.83659	-1.13035	-.88831	-.31267	-.88200
71	-.83659	.88028	-.35849	-1.74098	-.57759
72	-.83659	-1.13035	-.09358	.63955	1.12714
73	-.83659	.88028	-1.41813	-1.26488	-1.42995
74	-.83659	-1.13035	.17133	-1.02683	.36001
75	-.83659	-1.13035	1.49589	.16344	-1.42995
76	1.18938	.88028	-.62340	-.31267	-.15141
77	1.18938	.88028	1.23098	.63955	-1.42995
78	-.83659	-1.13035	-1.15322	-1.02683	1.12714
79	-.83659	-1.13035	-.62340	-.31267	-.79068
80	1.18938	-1.13035	.43625	1.82981	-.15141
81	-.83659	.88028	-.35849	-.78877	-.79068
82	1.18938	.88028	-1.41813	-1.74098	1.97950

	ZSco06	ZSco07	ZSco08	ZSco09	ZSco10
42	-.34842	1.73420	.80612	.62735	-.59710
43	-.34842	1.73420	.30884	.28313	-2.11391
44	-1.10611	2.03178	-.85148	-1.55267	1.29891
45	.40927	.06780	-.35420	1.43051	2.24691
46	-.34842	-1.06297	.88900	.74209	-1.73471
47	2.35762	1.02003	.47460	-.06108	.35090
48	-.45666	1.73420	.30884	-.06108	1.86771
49	-.56490	-1.36054	-.27132	-.06108	.35090
50	-.67314	.54392	.80612	.16840	1.86771
51	-1.10611	.48440	.64036	-.40529	-1.16590
52	-.34842	-1.83665	.22596	-1.32320	-.78670
53	-.02369	-.76539	.55748	-.40529	.91971
54	-.45666	-1.47957	.14308	1.31578	1.29891
55	-.88963	1.97226	.06020	.39787	-1.16590
56	-.56490	-1.71762	1.71780	1.88947	2.24691
57	.40927	1.49615	.47460	-.63477	-1.54510
58	.30103	-.34880	.80612	.62735	1.67811
59	.62575	-1.30102	.55748	.74209	2.43651
60	2.03289	1.02003	-.68572	-.29056	-.59710
61	.19279	-.88442	-.27132	-1.32320	.16130
62	-.56490	1.73420	-.76860	-.06108	-.78670
63	-.45666	-1.18199	.80612	-.40529	-.40750
64	-1.32259	1.19858	-1.68028	-1.66741	-.40750
65	-.56490	1.55566	.30884	-.63477	-.02830
66	-.99787	.00829	1.22052	.62735	-1.35550
67	1.27520	-.88442	.88900	-.29056	.73010
68	.08455	.12732	-1.92892	-1.66741	-.40750
69	1.27520	-.88442	-.51996	-.86424	-.78670
70	-.78138	.54392	-.27132	.85682	-.21790
71	.08455	.36538	.72324	.28313	1.10931
72	-.13194	.96052	.80612	.16840	.73010
73	.73400	-1.36054	.64036	-.29056	.54050
74	-.45666	.96052	-.93436	-1.32320	-.78670
75	1.92465	.00829	-.76860	-.06108	-.59710
76	1.59993	1.55566	-.27132	.05366	-1.16590
77	2.14113	-1.42005	1.46916	1.54525	.16130
78	-1.64732	-.28928	.06020	-.17582	-1.16590
79	-.99787	-1.36054	-2.34332	-1.89689	-.97630
80	-.88963	-.28928	-1.51452	-1.43793	-.78670
81	-1.10611	-.34880	-1.51452	-1.32320	.73010
82	-.56490	-.82491	.30884	-.17582	-.59710

	ZSco11	ZSco12	ZSco13	ZSco14	ZSco15
42	.74677	.17288	.31674	.48232	.46318
43	-1.25725	.66929	.31674	.48232	-.89231
44	.94717	-.81994	.31674	.48232	-.89231
45	1.94919	.17288	.90877	.48232	-.21456
46	-2.45966	1.16569	-1.45936	-.60155	-.89231
47	1.34798	1.16569	.31674	.48232	-.89231
48	.74677	.17288	1.50080	.48232	1.81867
49	.14557	1.16569	-1.45936	-.60155	-.89231
50	1.54838	.66929	.31674	.48232	-.89231
51	-.05484	.66929	-1.45936	-1.68541	-.89231
52	-1.05685	.17288	-1.45936	-4.93702	-.21456
53	.14557	-.81994	1.50080	.48232	-.89231
54	1.14758	-.81994	1.50080	.48232	-.89231
55	-1.45765	.66929	-.27530	-1.68541	.46318
56	1.94919	1.16569	-1.45936	-4.93702	-.89231
57	-.25524	.17288	.31674	-.60155	1.81867
58	1.34798	.17288	.90877	.48232	-.89231
59	1.94919	1.16569	.31674	-.60155	-.21456
60	-1.05685	.66929	-1.45936	.48232	-.89231
61	.34597	-1.31634	1.50080	.48232	1.14093
62	-.45564	.17288	.90877	.48232	1.81867
63	-1.25725	-.32353	.31674	-.60155	-.89231
64	-1.05685	-2.30916	-.27530	.48232	-.89231
65	-.05484	1.16569	1.50080	.48232	2.49642
66	-1.85846	.17288	.31674	.48232	.46318
67	.54637	.66929	.90877	.48232	1.81867
68	.74677	-1.81275	-.86733	.48232	-.89231
69	-.85645	.66929	-1.45936	.48232	-.21456
70	-.45564	1.16569	1.50080	.48232	-.89231
71	.74677	-.32353	-1.45936	.48232	-.89231
72	-1.05685	.66929	.31674	-1.68541	-.89231
73	-.05484	.66929	-.27530	-.60155	-.89231
74	.34597	.17288	-.86733	-.60155	-.89231
75	-.65604	.17288	-.86733	.48232	-.89231
76	-1.05685	-.81994	.31674	.48232	1.14093
77	-.05484	.66929	.90877	.48232	.46318
78	-1.05685	1.16569	-1.45936	-.60155	-.89231
79	-.85645	-2.30916	-1.45936	.48232	.
80	-.45564	-1.81275	.90877	.48232	.46318
81	1.14758	-.32353	-1.45936	.48232	-.89231
82	.34597	-.81994	-1.45936	.48232	-.89231

	ZSco16
42	.52614
43	.52614
44	.52614
45	.52614
46	-.45730
47	.52614
48	-.45730
49	.52614
50	.52614
51	-1.44075
52	-4.39109
53	.52614
54	.52614
55	-1.44075
56	-.45730
57	.52614
58	.52614
59	.52614
60	.52614
61	.52614
62	.52614
63	.52614
64	.52614
65	.52614
66	-.45730
67	.52614
68	.52614
69	.52614
70	.52614
71	-.45730
72	.52614
73	.52614
74	.52614
75	.52614
76	.52614
77	.52614
78	.52614
79	-.45730
80	-1.44075
81	-.45730
82	-.45730

	gender	PARENT_CYCLE_TRAINING_...	BIKEABILITY_L2_CODE	Can_Cycle	Cycle_Access	POSITIVE_ATTITUDE_CHILD	POSITIVE_ATTITUDE_PARENT	NEGATIVE_EXPERIENCES
83	Male	1	0	Yes	Yes	18	16	2.00
84	Female	0	0	Yes	No	14	11	.80
85	Female	0	1	Yes	Yes	27	27	1.00
86	Female	0	0	Yes	No	14	16	.80
87	Male	0	0	Yes	Yes	23	25	1.25
88	Female	0	0	No	Yes	17	18	1.75
89	Female	1	1	Yes	Yes	23	27	2.20
90	Male	1	1	Yes	Yes	18	19	1.25
91	Male	1	1	Yes	Yes	25	26	.29
92	Female	0	1	Yes	Yes	12	18	.40
93	Female	0	1	Yes	No	15	12	.50
94	Female	0	1	Yes	Yes	16	18	.00
95	Male	0	1	Yes	Yes	21	24	1.17
96	Prefer n	0	0	Yes	Yes	18	11	.29
97	Female	0	1	Yes	Yes	12	16	1.80
98	Female	0	0	No	No	15	16	1.00
99	Male	0	1	Yes	Yes	22	20	2.00
100	Female	1	1	Yes	Yes	18	23	1.29
101	Male	0	1	Yes	Yes	19	12	1.20
102	Prefer n	0	1	Yes	Yes	23	14	.83
103	Female	0	1	Yes	No	23	17	.80
104	Female	0	0	Yes	No	17	21	2.00
105	Male	1	0	Yes	No	22	22	1.00
106	Male	1	0	Yes	Yes	21	19	1.17
107	Male	0	1	Yes	No	21	18	.40
108	Female	0	1	Yes	Yes	15	18	1.40
109	Female	0	0	Yes	Yes	17	17	.00
110	Female	1	1	Yes	No	17	13	.86
111	Female	0	0	Yes	No	19	18	.60
112	Male	1	0	Yes	No	13	18	1.00
113	Female	0	1	Yes	Yes	20	14	.75
114	Female	1	1	Yes	No	19	13	1.80
115	Female	0	0	Yes	Yes	21	24	2.00
116	Male	1	1	Yes	Yes	18	12	.67
117	Female	1	1	Yes	Yes	18	12	2.80
118	Female	0	0	Yes	Yes	13	10	.67
119	Male	0	1	Yes	Yes	21	17	.88
120	Male	1	0	No	Yes	14	14	.88
121	Female	0	0	Yes	Yes	18	16	1.13
122	Male	0	0	Yes	Yes	22	26	.00
123	Male	0	1	Yes	Yes	23	19	.67

	MVPA	SEDENTARY	LIFE_SATISFACTION	WELLBEING	SATISFACTION_NOW	SATISFACTION_TEN_YEARS	Life_Satisfaction_Single_Item	Child_Cycling_Frequency
83	13	52	15	34	29	33	3.00	6.00
84	11	16	48	56	22	29	10.00	1.00
85	18	23	48	70	33	33	9.00	4.00
86	20	54	44	48	32	32	10.00	2.00
87	10	31	48	67	26	30	10.00	2.00
88	35	62	24	45	27	30	6.00	2.00
89	12	30	41	64	36	36	8.00	5.00
90	2	16	21	50	25	30	4.00	6.00
91	15	58	45	53	29	29	8.00	4.00
92	7	35	18	37	24	28	5.00	3.00
93	24	37	24	39	31	33	5.00	1.00
94	29	21	49	62	28	27	7.00	4.00
95	11	30	38	52	21	18	8.00	4.00
96	8	38	21	38	22	26	6.00	4.00
97	16	56	38	42	28	30	8.00	2.00
98	16	55	28	58	34	32	6.00	1.00
99	31	55	52	70	29	33	10.00	5.00
100	8	32	52	57	35	35	10.00	6.00
101	13	52	54	56	26	26	9.00	4.00
102	13	48	41	59	31	33	9.00	5.00
103	9	20	21	49	25	25	5.00	3.00
104	11	70	24	45	25	24	6.00	1.00
105	30	24	32	50	26	27	5.00	5.00
106	6	6	27	49	26	26	7.00	6.00
107	6	6	35	47	24	23	6.00	3.00
108	7	62	34	53	22	27	8.00	3.00
109	10	7	41	53	21	18	7.00	4.00
110	15	11	47	53	29	32	10.00	2.00
111	14	13	39	57	34	29	6.00	2.00
112	6	38	45	58	27	32	8.00	1.00
113	12	32	33	52	21	30	7.00	2.00
114	19	24	28	43	20	21	7.00	1.00
115	10	20	51	66	32	28	8.00	5.00
116	24	46	54	69	28	28	10.00	4.00
117	20	38	54	67	24	24	9.00	3.00
118	8	28	41	45	27	27	7.00	1.00
119	22	14	49	69	22	22	10.00	5.00
120	27	29	31	46	23	26	8.00	1.00
121	6	16	54	69	26	26	9.00	3.00
122	9	26	46	54	28	28	8.00	3.00
123	16	30	57	56	28	31	9.00	6.00

	Child_Walking_Frequency	Parent_Cycling_Frequency	Parent_Walking_Frequency	ZPARENT_CYCLE_TRAINING_CODE
83	6.00	4.00	6.00	1.20943
84	6.00	1.00	4.00	-.82281
85	6.00	4.00	6.00	-.82281
86	6.00	1.00	6.00	-.82281
87	4.00	1.00	3.00	-.82281
88	6.00	1.00	6.00	-.82281
89	5.00	5.00	5.00	1.20943
90	6.00	1.00	5.00	1.20943
91	6.00	4.00	6.00	1.20943
92	4.00	3.00	6.00	-.82281
93	6.00	1.00	5.00	-.82281
94	6.00	1.00	5.00	-.82281
95	6.00	6.00	5.00	-.82281
96	5.00	3.00	6.00	-.82281
97	6.00	3.00	5.00	-.82281
98	6.00	1.00	6.00	-.82281
99	6.00	1.00	6.00	-.82281
100	6.00	5.00	6.00	1.20943
101	6.00	1.00	6.00	-.82281
102	6.00	5.00	6.00	-.82281
103	5.00	1.00	3.00	-.82281
104	6.00	6.00	5.00	-.82281
105	6.00	3.00	6.00	1.20943
106	3.00	2.00	4.00	1.20943
107	5.00	1.00	6.00	-.82281
108	6.00	3.00	6.00	-.82281
109	5.00	5.00	5.00	-.82281
110	6.00	1.00	6.00	1.20943
111	6.00	3.00	6.00	-.82281
112	6.00	1.00	6.00	1.20943
113	6.00	1.00	6.00	-.82281
114	6.00	1.00	5.00	1.20943
115	6.00	6.00	5.00	-.82281
116	5.00	1.00	6.00	1.20943
117	6.00	3.00	6.00	1.20943
118	4.00	1.00	4.00	-.82281
119	6.00	1.00	6.00	-.82281
120	3.00	1.00	3.00	1.20943
121	4.00	1.00	4.00	-.82281
122	5.00	2.00	4.00	-.82281
123	6.00	3.00	6.00	-.82281

	ZBIKEABILITY_L2_CODE	ZPOSITIVE_ATTITUDE_CHILD	ZPOSITIVE_ATTITUDE_PARENT
83	-1.12763	-.09509	-.31978
84	-1.12763	-1.16320	-1.51606
85	.88249	2.30816	2.31201
86	-1.12763	-1.16320	-.31978
87	-1.12763	1.24005	1.83351
88	-1.12763	-.36212	.15872
89	.88249	1.24005	2.31201
90	.88249	-.09509	.39798
91	.88249	1.77410	2.07276
92	.88249	-1.69725	.15872
93	.88249	-.89617	-1.27680
94	.88249	-.62914	.15872
95	.88249	.70599	1.59425
96	-1.12763	-.09509	-1.51606
97	.88249	-1.69725	-.31978
98	-1.12763	-.89617	-.31978
99	.88249	.97302	.63723
100	.88249	-.09509	1.35500
101	.88249	.17194	-1.27680
102	.88249	1.24005	-.79829
103	.88249	1.24005	-.08053
104	-1.12763	-.36212	.87649
105	-1.12763	.97302	1.11574
106	-1.12763	.70599	.39798
107	.88249	.70599	.15872
108	.88249	-.89617	.15872
109	-1.12763	-.36212	-.08053
110	.88249	-.36212	-1.03755
111	-1.12763	.17194	.15872
112	-1.12763	-1.43022	.15872
113	.88249	.43897	-.79829
114	.88249	.17194	-1.03755
115	-1.12763	.70599	1.59425
116	.88249	-.09509	-1.27680
117	.88249	-.09509	-1.27680
118	-1.12763	-1.43022	-1.75531
119	.88249	.70599	-.08053
120	-1.12763	-1.16320	-.79829
121	-1.12763	-.09509	-.31978
122	-1.12763	.97302	2.07276
123	.88249	1.24005	.39798

	ZNEGATIVE_EXPERIENCES	ZMVPA	ZSEDENTARY	ZLIFE_SATISFACTION
83	1.12151	-.35051	.96984	-1.96645
84	-.41821	-.56825	-1.17182	.73467
85	-.16159	.19384	-.75539	.73467
86	-.41821	.41158	1.08882	.40726
87	.15918	-.67712	-.27946	.73467
88	.80073	2.04464	1.56475	-1.22978
89	1.37813	-.45938	-.33895	.16171
90	.15918	-1.54809	-1.17182	-1.47533
91	-1.07810	-.13277	1.32679	.48912
92	-.93146	-1.00373	-.04150	-1.72089
93	-.80315	.84707	.07748	-1.22978
94	-1.44470	1.39142	-.87437	.81652
95	.05226	-.56825	-.33895	-.08385
96	-1.07810	-.89486	.13697	-1.47533
97	.86489	-.02390	1.20780	-.08385
98	-.16159	-.02390	1.14831	-.90237
99	1.12151	1.60916	1.14831	1.06208
100	.20501	-.89486	-.21997	1.06208
101	.09503	-.35051	.96984	1.22578
102	-.37544	-.35051	.73188	.16171
103	-.41821	-.78599	-.93386	-1.47533
104	1.12151	-.56825	2.04067	-1.22978
105	-.16159	1.50029	-.69589	-.57496
106	.05226	-1.11260	-1.76673	-.98422
107	-.93146	-1.11260	-1.76673	-.32940
108	.35165	-1.00373	1.56475	-.41126
109	-1.44470	-.67712	-1.70724	.16171
110	-.34489	-.13277	-1.46927	.65282
111	-.67483	-.24164	-1.35029	-.00200
112	-.16159	-1.11260	.13697	.48912
113	-.48237	-.45938	-.21997	-.49311
114	.86489	.30271	-.69589	-.90237
115	1.12151	-.67712	-.93386	.98023
116	-.58929	.84707	.61290	1.22578
117	2.14799	.41158	.13697	1.22578
118	-.58929	-.89486	-.45793	.16171
119	-.32198	.62932	-1.29080	.81652
120	-.32198	1.17368	-.39844	-.65681
121	-.00121	-1.11260	-1.17182	1.22578
122	-1.44470	-.78599	-.57691	.57097
123	-.58929	-.02390	-.33895	1.47134

	ZWELLBEING	ZSATISFACTION_NOW	ZSATISFACTION_TEN_YEARS
83	-2.36902	.35074	.91776
84	.15861	-.95995	.14905
85	1.76710	1.09970	.91776
86	-.76053	.91246	.72558
87	1.42242	-.21099	.34123
88	-1.10521	-.02375	.34123
89	1.07774	1.66143	1.49429
90	-.53075	-.39823	.34123
91	-.18607	.35074	.14905
92	-2.02434	-.58547	-.04312
93	-1.79456	.72522	.91776
94	.84796	.16349	-.23530
95	-.30096	-1.14720	-1.96489
96	-1.90945	-.95995	-.42748
97	-1.44988	.16349	.34123
98	.38839	1.28694	.72558
99	1.76710	.35074	.91776
100	.27350	1.47418	1.30211
101	.15861	-.21099	-.42748
102	.50328	.72522	.91776
103	-.64564	-.39823	-.61965
104	-1.10521	-.39823	-.81183
105	-.53075	-.21099	-.23530
106	-.64564	-.21099	-.42748
107	-.87542	-.58547	-1.00401
108	-.18607	-.95995	-.23530
109	-.18607	-1.14720	-1.96489
110	-.18607	.35074	.72558
111	.27350	1.28694	.14905
112	.38839	-.02375	.72558
113	-.30096	-1.14720	.34123
114	-1.33499	-1.33444	-1.38836
115	1.30753	.91246	-.04312
116	1.65220	.16349	-.04312
117	1.42242	-.58547	-.81183
118	-1.10521	-.02375	-.23530
119	1.65220	-.95995	-1.19618
120	-.99031	-.77271	-.42748
121	1.65220	-.21099	-.42748
122	-.07118	.16349	-.04312
123	.15861	.16349	.53341

	ZLife_Satisfaction_Single_Item	ZChild_Cycling_Frequency
83	-2.27369	1.50175
84	1.15357	-1.44967
85	.66396	.32118
86	1.15357	-.85939
87	1.15357	-.85939
88	-.80487	-.85939
89	.17435	.91147
90	-1.78409	1.50175
91	.17435	.32118
92	-1.29448	-.26910
93	-1.29448	-1.44967
94	-.31526	.32118
95	.17435	.32118
96	-.80487	.32118
97	.17435	-.85939
98	-.80487	-1.44967
99	1.15357	.91147
100	1.15357	1.50175
101	.66396	.32118
102	.66396	.91147
103	-1.29448	-.26910
104	-.80487	-1.44967
105	-1.29448	.91147
106	-.31526	1.50175
107	-.80487	-.26910
108	.17435	-.26910
109	-.31526	.32118
110	1.15357	-.85939
111	-.80487	-.85939
112	.17435	-1.44967
113	-.31526	-.85939
114	-.31526	-1.44967
115	.17435	.91147
116	1.15357	.32118
117	.66396	-.26910
118	-.31526	-1.44967
119	1.15357	.91147
120	.17435	-1.44967
121	.66396	-.26910
122	.17435	-.26910
123	.66396	1.50175

	ZChild_Walking_Frequency	ZParent_Cycling_Frequency
83	.48168	1.15468
84	.48168	-.88951
85	.48168	1.15468
86	.48168	-.88951
87	-1.70194	-.88951
88	.48168	-.88951
89	-.61013	1.83607
90	.48168	-.88951
91	.48168	1.15468
92	-1.70194	.47328
93	.48168	-.88951
94	.48168	-.88951
95	.48168	2.51747
96	-.61013	.47328
97	.48168	.47328
98	.48168	-.88951
99	.48168	-.88951
100	.48168	1.83607
101	.48168	-.88951
102	.48168	1.83607
103	-.61013	-.88951
104	.48168	2.51747
105	.48168	.47328
106	-2.79376	-.20811
107	-.61013	-.88951
108	.48168	.47328
109	-.61013	1.83607
110	.48168	-.88951
111	.48168	.47328
112	.48168	-.88951
113	.48168	-.88951
114	.48168	-.88951
115	.48168	2.51747
116	-.61013	-.88951
117	.48168	.47328
118	-1.70194	-.88951
119	.48168	-.88951
120	-2.79376	-.88951
121	-1.70194	-.88951
122	-.61013	-.20811
123	.48168	.47328

	ZParent_Walking_Frequency	MAH_2	Probability_MAH_2	filter_$
83	.52943	8.98688	.4385	Selected
84	-1.45230	8.97629	.4395	Selected
85	.52943	8.91360	.4453	Selected
86	.52943	8.85728	.4506	Selected
87	-2.44316	8.82035	.4540	Selected
88	.52943	8.76601	.4591	Selected
89	-.46143	8.75721	.4600	Selected
90	-.46143	8.75704	.4600	Selected
91	.52943	8.62193	.4729	Selected
92	.52943	8.55162	.4796	Selected
93	-.46143	8.51618	.4831	Selected
94	-.46143	8.40212	.4942	Selected
95	-.46143	8.35210	.4991	Selected
96	.52943	8.34889	.4994	Selected
97	-.46143	8.29216	.5050	Selected
98	.52943	8.22492	.5116	Selected
99	.52943	8.20205	.5139	Selected
100	.52943	8.15206	.5189	Selected
101	.52943	8.05650	.5285	Selected
102	.52943	7.99850	.5343	Selected
103	-2.44316	7.92903	.5413	Selected
104	-.46143	7.86832	.5475	Selected
105	.52943	7.85887	.5484	Selected
106	-1.45230	7.79052	.5554	Selected
107	.52943	7.72210	.5624	Selected
108	.52943	7.54228	.5808	Selected
109	-.46143	7.48969	.5863	Selected
110	.52943	7.48809	.5864	Selected
111	.52943	7.35709	.6000	Selected
112	.52943	7.34341	.6014	Selected
113	.52943	7.33922	.6018	Selected
114	-.46143	7.31011	.6049	Selected
115	-.46143	7.11840	.6248	Selected
116	.52943	7.07959	.6288	Selected
117	.52943	7.05950	.6309	Selected
118	-1.45230	7.03074	.6339	Selected
119	.52943	6.79545	.6584	Selected
120	-2.44316	6.77730	.6603	Selected
121	-1.45230	6.71192	.6671	Selected
122	-1.45230	6.66639	.6718	Selected
123	.52943	6.53368	.6855	Selected

	ZSco01	ZSco02	ZSco03	ZSco04	ZSco05
83	1.18938	-1.13035	-.09358	-.31267	1.12714
84	-.83659	-1.13035	-1.15322	-1.50293	-.40712
85	-.83659	.88028	2.29062	2.30592	-.15141
86	-.83659	-1.13035	-1.15322	-.31267	-.40712
87	-.83659	-1.13035	1.23098	1.82981	.16823
88	-.83659	-1.13035	-.35849	.16344	.80750
89	1.18938	.88028	1.23098	2.30592	1.38285
90	1.18938	.88028	-.09358	.40149	.16823
91	1.18938	.88028	1.76080	2.06786	-1.06465
92	-.83659	.88028	-1.68304	.16344	-.91853
93	-.83659	.88028	-.88831	-1.26488	-.79068
94	-.83659	.88028	-.62340	.16344	-1.42995
95	-.83659	.88028	.70116	1.59176	.06168
96	-.83659	-1.13035	-.09358	-1.50293	-1.06465
97	-.83659	.88028	-1.68304	-.31267	.87143
98	-.83659	-1.13035	-.88831	-.31267	-.15141
99	-.83659	.88028	.96607	.63955	1.12714
100	1.18938	.88028	-.09358	1.35370	.21389
101	-.83659	.88028	.17133	-1.26488	.10430
102	-.83659	.88028	1.23098	-.78877	-.36450
103	-.83659	.88028	1.23098	-.07461	-.40712
104	-.83659	-1.13035	-.35849	.87760	1.12714
105	1.18938	-1.13035	.96607	1.11565	-.15141
106	1.18938	-1.13035	.70116	.40149	.06168
107	-.83659	.88028	.70116	.16344	-.91853
108	-.83659	.88028	-.88831	.16344	.36001
109	-.83659	-1.13035	-.35849	-.07461	-1.42995
110	1.18938	.88028	-.35849	-1.02683	-.33406
111	-.83659	-1.13035	.17133	.16344	-.66283
112	1.18938	-1.13035	-1.41813	.16344	-.15141
113	-.83659	.88028	.43625	-.78877	-.47104
114	1.18938	.88028	.17133	-1.02683	.87143
115	-.83659	-1.13035	.70116	1.59176	1.12714
116	1.18938	.88028	-.09358	-1.26488	-.57759
117	1.18938	.88028	-.09358	-1.26488	2.14997
118	-.83659	-1.13035	-1.41813	-1.74098	-.57759
119	-.83659	.88028	.70116	-.07461	-.31123
120	1.18938	-1.13035	-1.15322	-.78877	-.31123
121	-.83659	-1.13035	-.09358	-.31267	.00841
122	-.83659	-1.13035	.96607	2.06786	-1.42995
123	-.83659	.88028	1.23098	.40149	-.57759

	ZSco06	ZSco07	ZSco08	ZSco09	ZSco10
83	-.34842	.96052	-2.01180	-2.35584	.35090
84	-.56490	-1.18199	.72324	.16840	-.97630
85	.19279	-.76539	.72324	1.77473	1.10931
86	.40927	1.07955	.39172	-.74951	.91971
87	-.67314	-.28928	.72324	1.43051	-.21790
88	2.03289	1.55566	-1.26588	-1.09372	-.02830
89	-.45666	-.34880	.14308	1.08630	1.67811
90	-1.53907	-1.18199	-1.51452	-.52003	-.40750
91	-.13194	1.31760	.47460	-.17582	.35090
92	-.99787	-.05122	-1.76316	-2.01162	-.59710
93	.84224	.06780	-1.26588	-1.78215	.73010
94	1.38344	-.88442	.80612	.85682	.16130
95	-.56490	-.34880	-.10556	-.29056	-1.16590
96	-.88963	.12732	-1.51452	-1.89689	-.97630
97	-.02369	1.19858	-.10556	-1.43793	.16130
98	-.02369	1.13906	-.93436	.39787	1.29891
99	1.59993	1.13906	1.05476	1.77473	.35090
100	-.88963	-.22977	1.05476	.28313	1.48851
101	-.34842	.96052	1.22052	.16840	-.21790
102	-.34842	.72246	.14308	.51261	.73010
103	-.78138	-.94394	-1.51452	-.63477	-.40750
104	-.56490	2.03178	-1.26588	-1.09372	-.40750
105	1.49169	-.70588	-.60284	-.52003	-.21790
106	-1.10611	-1.77714	-1.01724	-.63477	-.21790
107	-1.10611	-1.77714	-.35420	-.86424	-.59710
108	-.99787	1.55566	-.43708	-.17582	-.97630
109	-.67314	-1.71762	.14308	-.17582	-1.16590
110	-.13194	-1.47957	.64036	-.17582	.35090
111	-.24018	-1.36054	-.02268	.28313	1.29891
112	-1.10611	.12732	.47460	.39787	-.02830
113	-.45666	-.22977	-.51996	-.29056	-1.16590
114	.30103	-.70588	-.93436	-1.32320	-1.35550
115	-.67314	-.94394	.97188	1.31578	.91971
116	.84224	.60343	1.22052	1.65999	.16130
117	.40927	.12732	1.22052	1.43051	-.59710
118	-.88963	-.46782	.14308	-1.09372	-.02830
119	.62575	-1.30102	.80612	1.65999	-.97630
120	1.16696	-.40831	-.68572	-.97898	-.78670
121	-1.10611	-1.18199	1.22052	1.65999	-.21790
122	-.78138	-.58685	.55748	-.06108	.16130
123	-.02369	-.34880	1.46916	.16840	.16130

	ZSco11	ZSco12	ZSco13	ZSco14	ZSco15
83	.94717	-2.30916	1.50080	.48232	1.14093
84	.14557	1.16569	-1.45936	.48232	-.89231
85	.94717	.66929	.31674	.48232	1.14093
86	.74677	1.16569	-.86733	.48232	-.89231
87	.34597	1.16569	-.86733	-1.68541	-.89231
88	.34597	-.81994	-.86733	.48232	-.89231
89	1.54838	.17288	.90877	-.60155	1.81867
90	.34597	-1.81275	1.50080	.48232	-.89231
91	.14557	.17288	.31674	.48232	1.14093
92	-.05484	-1.31634	-.27530	-1.68541	.46318
93	.94717	-1.31634	-1.45936	.48232	-.89231
94	-.25524	-.32353	.31674	.48232	-.89231
95	-2.05886	.17288	.31674	.48232	2.49642
96	-.45564	-.81994	.31674	-.60155	.46318
97	.34597	.17288	-.86733	.48232	.46318
98	.74677	-.81994	-1.45936	.48232	-.89231
99	.94717	1.16569	.90877	.48232	-.89231
100	1.34798	1.16569	1.50080	.48232	1.81867
101	-.45564	.66929	.31674	.48232	-.89231
102	.94717	.66929	.90877	.48232	1.81867
103	-.65604	-1.31634	-.27530	-.60155	-.89231
104	-.85645	-.81994	-1.45936	.48232	2.49642
105	-.25524	-1.31634	.90877	.48232	.46318
106	-.45564	-.32353	1.50080	-2.76928	-.21456
107	-1.05685	-.81994	-.27530	-.60155	-.89231
108	-.25524	.17288	-.27530	.48232	.46318
109	-2.05886	-.32353	.31674	-.60155	1.81867
110	.74677	1.16569	-.86733	.48232	-.89231
111	.14557	-.81994	-.86733	.48232	.46318
112	.74677	.17288	-1.45936	.48232	-.89231
113	.34597	-.32353	-.86733	.48232	-.89231
114	-1.45765	-.32353	-1.45936	.48232	-.89231
115	-.05484	.17288	.90877	.48232	2.49642
116	-.05484	1.16569	.31674	-.60155	-.89231
117	-.85645	.66929	-.27530	.48232	.46318
118	-.25524	-.32353	-1.45936	-1.68541	-.89231
119	-1.25725	1.16569	.90877	.48232	-.89231
120	-.45564	.17288	-1.45936	-2.76928	-.89231
121	-.45564	.66929	-.27530	-1.68541	-.89231
122	-.05484	.17288	-.27530	-.60155	-.21456
123	.54637	.66929	1.50080	.48232	.46318

	ZSco16
83	.52614
84	-1.44075
85	.52614
86	.52614
87	-2.42420
88	.52614
89	-.45730
90	-.45730
91	.52614
92	.52614
93	-.45730
94	-.45730
95	-.45730
96	.52614
97	-.45730
98	.52614
99	.52614
100	.52614
101	.52614
102	.52614
103	-2.42420
104	-.45730
105	.52614
106	-1.44075
107	.52614
108	.52614
109	-.45730
110	.52614
111	.52614
112	.52614
113	.52614
114	-.45730
115	-.45730
116	.52614
117	.52614
118	-1.44075
119	.52614
120	-2.42420
121	-1.44075
122	-1.44075
123	.52614

	gender	PARENT_CYCLE_TRAINING_...	BIKEABILITY_L2_CODE	Can_Cycle	Cycle_Access	POSITIVE_ATTITUDE_CHILD	POSITIVE_ATTITUDE_PARENT	NEGATIVE_EXPERIENCES
124	Male	1	0	Yes	Yes	14	17	.20
125	Male	1	1	Yes	Yes	14	13	1.00
126	Male	1	1	Yes	Yes	22	23	1.00
127	Male	0	0	Yes	No	18	16	1.60
128	Male	0	0	Yes	Yes	14	13	.50
129	Male	1	1	Yes	Yes	24	21	.17
130	Female	0	0	Yes	Yes	25	21	.11
131	Female	0	1	Yes	No	22	21	.17
132	Male	0	1	Yes	Yes	19	20	1.00
133	Male	0	0	Yes	No	21	16	1.00
134	Female	0	1	Yes	Yes	20	15	.00
135	Male	0	0	Yes	Yes	24	17	1.00
136	Male	0	1	Yes	Yes	19	16	.80
137	Female	1	1	Yes	Yes	17	19	1.20
138	Female	1	1	Yes	Yes	20	12	.83
139	Male	1	1	Yes	Yes	24	18	.50
140	Female	1	1	Yes	Yes	17	20	.60
141	Male	0	1	Yes	Yes	21	15	2.17
142	Female	0	0	Yes	Yes	18	17	1.00
143	Male	1	0	No	No	17	19	2.17
144	Male	0	1	Yes	Yes	24	18	1.17
145	Female	1	1	Yes	Yes	16	14	1.00
146	Male	0	1	Yes	Yes	19	20	1.50
147	Female	1	1	Yes	Yes	18	18	.40
148	Male	0	0	Yes	Yes	14	13	2.20
149	Female	0	1	Yes	Yes	19	15	1.00
150	Female	1	0	Yes	No	18	17	1.00
151	Male	1	1	Yes	No	13	12	1.20
152	Male	1	1	Yes	No	19	14	.75
153	Male	0	1	Yes	No	16	15	1.00
154	Female	1	0	No	No	17	17	1.00
155	Male	1	1	Yes	Yes	15	17	.20
156	Female	0	0	Yes	Yes	15	15	.43
157	Female	0	0	Yes	No	19	18	.00
158	Female	1	1	Yes	No	21	16	1.25
159	Female	0	0	Yes	No	12	13	1.60
160	Male	0	1	Yes	Yes	18	16	2.13
161	Male	0	0	Yes	No	17	12	3.00
162	Male	0	0	No	Yes	17	16	.29
163	Female	0	1	Yes	Yes	13	16	2.17
164	Female	0	0	Yes	No	16	15	.00

	MVPA	SEDENTARY	LIFE_SATISFACTION	WELLBEING	SATISFACTION_NOW	SATISFACTION_TEN_YEARS	Life_Satisfaction_Single_Item	Child_Cycling_Frequency
124	1	27	28	43	24	27	7.00	3.00
125	3	21	28	53	25	28	5.00	4.00
126	13	40	44	51	35	37	8.00	6.00
127	13	21	31	43	20	20	5.00	1.00
128	5	55	39	56	23	23	8.00	3.00
129	31	36	52	62	31	27	10.00	4.00
130	23	43	37	55	32	32	6.00	3.00
131	8	13	46	67	32	32	8.00	2.00
132	8	44	45	61	25	23	7.00	5.00
133	19	48	41	55	19	22	7.00	4.00
134	13	39	43	63	31	34	7.00	3.00
135	19	37	50	64	31	31	8.00	6.00
136	20	51	32	54	32	32	9.00	6.00
137	16	58	43	60	28	26	7.00	5.00
138	12	24	35	54	25	27	6.00	5.00
139	14	43	37	56	28	28	9.00	5.00
140	6	40	32	47	33	36	7.00	4.00
141	24	32	41	60	20	21	9.00	6.00
142	19	73	38	53	26	26	8.00	4.00
143	14	15	50	60	22	22	10.00	1.00
144	16	37	52	65	33	35	9.00	5.00
145	12	33	45	66	33	32	9.00	5.00
146	25	35	57	67	36	36	10.00	5.00
147	7	50	54	64	31	30	10.00	3.00
148	18	60	36	48	31	31	8.00	4.00
149	19	27	56	69	34	35	10.00	4.00
150	22	28	45	51	29	34	7.00	2.00
151	15	50	47	55	31	31	9.00	1.00
152	22	42	43	51	27	33	9.00	2.00
153	5	47	21	41	24	29	4.00	2.00
154	17	21	40	60	26	27	10.00	1.00
155	9	40	54	63	30	30	9.00	4.00
156	6	41	37	49	29	29	9.00	4.00
157	13	4	51	62	32	32	9.00	5.00
158	9	38	50	62	34	35	9.00	2.00
159	9	26	32	44	26	27	7.00	3.00
160	15	16	48	57	26	22	8.00	6.00
161	9	40	45	53	21	24	8.00	.
162	7	20	47	52	21	24	8.00	1.00
163	22	33	46	59	27	28	8.00	5.00
164	24	20	53	64	31	32	10.00	1.00

	Child_Walking_Frequency	Parent_Cycling_Frequency	Parent_Walking_Frequency	ZPARENT_CYCLE_TRAINING_CODE
124	4.00	2.00	4.00	1.20943
125	6.00	3.00	6.00	1.20943
126	5.00	4.00	6.00	1.20943
127	6.00	1.00	4.00	-.82281
128	6.00	1.00	6.00	-.82281
129	6.00	4.00	5.00	1.20943
130	5.00	3.00	6.00	-.82281
131	6.00	1.00	6.00	-.82281
132	6.00	2.00	6.00	-.82281
133	6.00	3.00	6.00	-.82281
134	6.00	3.00	6.00	-.82281
135	6.00	3.00	6.00	-.82281
136	6.00	3.00	5.00	-.82281
137	6.00	5.00	6.00	1.20943
138	6.00	3.00	5.00	1.20943
139	6.00	3.00	5.00	1.20943
140	6.00	4.00	5.00	1.20943
141	6.00	4.00	6.00	-.82281
142	5.00	1.00	5.00	-.82281
143	6.00	4.00	6.00	1.20943
144	5.00	3.00	4.00	-.82281
145	6.00	3.00	5.00	1.20943
146	6.00	1.00	6.00	-.82281
147	6.00	3.00	6.00	1.20943
148	6.00	3.00	6.00	-.82281
149	6.00	1.00	6.00	-.82281
150	6.00	1.00	6.00	1.20943
151	6.00	1.00	6.00	1.20943
152	6.00	1.00	6.00	1.20943
153	6.00	1.00	6.00	-.82281
154	6.00	1.00	6.00	1.20943
155	5.00	3.00	5.00	1.20943
156	6.00	1.00	6.00	-.82281
157	6.00	4.00	6.00	-.82281
158	6.00	1.00	6.00	1.20943
159	6.00	1.00	6.00	-.82281
160	6.00	3.00	6.00	-.82281
161	.	.	.	-.82281
162	6.00	1.00	5.00	-.82281
163	5.00	3.00	6.00	-.82281
164	6.00	1.00	6.00	-.82281

	ZBIKEABILITY_L2_CODE	ZPOSITIVE_ATTITUDE_CHILD	ZPOSITIVE_ATTITUDE_PARENT
124	-1.12763	-1.16320	-.08053
125	.88249	-1.16320	-1.03755
126	.88249	.97302	1.35500
127	-1.12763	-.09509	-.31978
128	-1.12763	-1.16320	-1.03755
129	.88249	1.50708	.87649
130	-1.12763	1.77410	.87649
131	.88249	.97302	.87649
132	.88249	.17194	.63723
133	-1.12763	.70599	-.31978
134	.88249	.43897	-.55904
135	-1.12763	1.50708	-.08053
136	.88249	.17194	-.31978
137	.88249	-.36212	.39798
138	.88249	.43897	-1.27680
139	.88249	1.50708	.15872
140	.88249	-.36212	.63723
141	.88249	.70599	-.55904
142	-1.12763	-.09509	-.08053
143	-1.12763	-.36212	.39798
144	.88249	1.50708	.15872
145	.88249	-.62914	-.79829
146	.88249	.17194	.63723
147	.88249	-.09509	.15872
148	-1.12763	-1.16320	-1.03755
149	.88249	.17194	-.55904
150	-1.12763	-.09509	-.08053
151	.88249	-1.43022	-1.27680
152	.88249	.17194	-.79829
153	.88249	-.62914	-.55904
154	-1.12763	-.36212	-.08053
155	.88249	-.89617	-.08053
156	-1.12763	-.89617	-.55904
157	-1.12763	.17194	.15872
158	.88249	.70599	-.31978
159	-1.12763	-1.69725	-1.03755
160	.88249	-.09509	-.31978
161	-1.12763	-.36212	-1.27680
162	-1.12763	-.36212	-.31978
163	.88249	-1.43022	-.31978
164	-1.12763	-.62914	-.55904

	ZNEGATIVE_EXPERIENCES	ZMVPA	ZSEDENTARY	ZLIFE_SATISFACTION
124	-1.18808	-1.65696	-.51742	-.90237
125	-.16159	-1.43922	-.87437	-.90237
126	-.16159	-.35051	.25595	.40726
127	.60827	-.35051	-.87437	-.65681
128	-.80315	-1.22147	1.14831	-.00200
129	-1.23085	1.60916	.01799	1.06208
130	-1.30213	.73820	.43443	-.16570
131	-1.23085	-.89486	-1.35029	.57097
132	-.16159	-.89486	.49392	.48912
133	-.16159	.30271	.73188	.16171
134	-1.44470	-.35051	.19646	.32541
135	-.16159	.30271	.07748	.89838
136	-.41821	.41158	.91035	-.57496
137	.09503	-.02390	1.32679	.32541
138	-.37544	-.45938	-.69589	-.32940
139	-.80315	-.24164	.43443	-.16570
140	-.67483	-1.11260	.25595	-.57496
141	1.33536	.84707	-.21997	.16171
142	-.16159	.30271	2.21914	-.08385
143	1.33536	-.24164	-1.23131	.89838
144	.05226	-.02390	.07748	1.06208
145	-.16159	-.45938	-.16048	.48912
146	.47996	.95594	-.04150	1.47134
147	-.93146	-1.00373	.85086	1.22578
148	1.37813	.19384	1.44577	-.24755
149	-.16159	.30271	-.51742	1.38949
150	-.16159	.62932	-.45793	.48912
151	.09503	-.13277	.85086	.65282
152	-.48237	.62932	.37494	.32541
153	-.16159	-1.22147	.67239	-1.47533
154	-.16159	.08497	-.87437	.07986
155	-1.18808	-.78599	.25595	1.22578
156	-.89480	-1.11260	.31545	-.16570
157	-1.44470	-.35051	-1.88571	.98023
158	.15918	-.78599	.13697	.89838
159	.60827	-.78599	-.57691	-.57496
160	1.28190	-.13277	-1.17182	.73467
161	2.40461	-.78599	.25595	.48912
162	-1.07810	-1.00373	-.93386	.65282
163	1.33536	.62932	-.16048	.57097
164	-1.44470	.84707	-.93386	1.14393

	ZWELLBEING	ZSATISFACTION_NOW	ZSATISFACTION_TEN_YEARS
124	-1.33499	-.58547	-.23530
125	-.18607	-.39823	-.04312
126	-.41585	1.47418	1.68647
127	-1.33499	-1.33444	-1.58054
128	.15861	-.77271	-1.00401
129	.84796	.72522	-.23530
130	.04372	.91246	.72558
131	1.42242	.91246	.72558
132	.73307	-.39823	-1.00401
133	.04372	-1.52168	-1.19618
134	.96285	.72522	1.10994
135	1.07774	.72522	.53341
136	-.07118	.91246	.72558
137	.61818	.16349	-.42748
138	-.07118	-.39823	-.23530
139	.15861	.16349	-.04312
140	-.87542	1.09970	1.49429
141	.61818	-1.33444	-1.38836
142	-.18607	-.21099	-.42748
143	.61818	-.95995	-1.19618
144	1.19264	1.09970	1.30211
145	1.30753	1.09970	.72558
146	1.42242	1.66143	1.49429
147	1.07774	.72522	.34123
148	-.76053	.72522	.53341
149	1.65220	1.28694	1.30211
150	-.41585	.35074	1.10994
151	.04372	.72522	.53341
152	-.41585	-.02375	.91776
153	-1.56477	-.58547	.14905
154	.61818	-.21099	-.23530
155	.96285	.53798	.34123
156	-.64564	.35074	.14905
157	.84796	.91246	.72558
158	.84796	1.28694	1.30211
159	-1.22010	-.21099	-.23530
160	.27350	-.21099	-1.19618
161	-.18607	-1.14720	-.81183
162	-.30096	-1.14720	-.81183
163	.50328	-.02375	-.04312
164	1.07774	.72522	.72558

	ZLife_Satisfaction_Single_Item	ZChild_Cycling_Frequency
124	-.31526	-.26910
125	-1.29448	.32118
126	.17435	1.50175
127	-1.29448	-1.44967
128	.17435	-.26910
129	1.15357	.32118
130	-.80487	-.26910
131	.17435	-.85939
132	-.31526	.91147
133	-.31526	.32118
134	-.31526	-.26910
135	.17435	1.50175
136	.66396	1.50175
137	-.31526	.91147
138	-.80487	.91147
139	.66396	.91147
140	-.31526	.32118
141	.66396	1.50175
142	.17435	.32118
143	1.15357	-1.44967
144	.66396	.91147
145	.66396	.91147
146	1.15357	.91147
147	1.15357	-.26910
148	.17435	.32118
149	1.15357	.32118
150	-.31526	-.85939
151	.66396	-1.44967
152	.66396	-.85939
153	-1.78409	-.85939
154	1.15357	-1.44967
155	.66396	.32118
156	.66396	.32118
157	.66396	.91147
158	.66396	-.85939
159	-.31526	-.26910
160	.17435	1.50175
161	.17435	.
162	.17435	-1.44967
163	.17435	.91147
164	1.15357	-1.44967

	ZChild_Walking_Frequency	ZParent_Cycling_Frequency
124	-1.70194	-.20811
125	.48168	.47328
126	-.61013	1.15468
127	.48168	-.88951
128	.48168	-.88951
129	.48168	1.15468
130	-.61013	.47328
131	.48168	-.88951
132	.48168	-.20811
133	.48168	.47328
134	.48168	.47328
135	.48168	.47328
136	.48168	.47328
137	.48168	1.83607
138	.48168	.47328
139	.48168	.47328
140	.48168	1.15468
141	.48168	1.15468
142	-.61013	-.88951
143	.48168	1.15468
144	-.61013	.47328
145	.48168	.47328
146	.48168	-.88951
147	.48168	.47328
148	.48168	.47328
149	.48168	-.88951
150	.48168	-.88951
151	.48168	-.88951
152	.48168	-.88951
153	.48168	-.88951
154	.48168	-.88951
155	-.61013	.47328
156	.48168	-.88951
157	.48168	1.15468
158	.48168	-.88951
159	.48168	-.88951
160	.48168	.47328
161	.	.
162	.48168	-.88951
163	-.61013	.47328
164	.48168	-.88951

	ZParent_Walking_Frequency	MAH_2	Probability_MAH_2	filter_$
124	-1.45230	6.50731	.6883	Selected
125	.52943	6.44215	.6950	Selected
126	.52943	6.40295	.6990	Selected
127	-1.45230	6.37362	.7020	Selected
128	.52943	6.32000	.7075	Selected
129	-.46143	6.27033	.7126	Selected
130	.52943	6.20335	.7194	Selected
131	.52943	6.09499	.7304	Selected
132	.52943	6.06388	.7335	Selected
133	.52943	5.92997	.7469	Selected
134	.52943	5.82767	.7570	Selected
135	.52943	5.82593	.7572	Selected
136	-.46143	5.76588	.7631	Selected
137	.52943	5.76232	.7635	Selected
138	-.46143	5.71429	.7681	Selected
139	-.46143	5.70335	.7692	Selected
140	-.46143	5.66416	.7730	Selected
141	.52943	5.59765	.7794	Selected
142	-.46143	5.56092	.7829	Selected
143	.52943	5.52974	.7859	Selected
144	-1.45230	5.51486	.7873	Selected
145	-.46143	5.41449	.7968	Selected
146	.52943	5.31059	.8064	Selected
147	.52943	5.27500	.8097	Selected
148	.52943	5.21876	.8148	Selected
149	.52943	5.17363	.8189	Selected
150	.52943	5.15749	.8204	Selected
151	.52943	5.13676	.8222	Selected
152	.52943	5.12429	.8233	Selected
153	.52943	5.11855	.8239	Selected
154	.52943	5.04236	.8306	Selected
155	-.46143	4.96070	.8377	Selected
156	.52943	4.94312	.8392	Selected
157	.52943	4.92685	.8406	Selected
158	.52943	4.91920	.8413	Selected
159	.52943	4.91200	.8419	Selected
160	.52943	4.91167	.8419	Selected
161	.	4.73951	.8564	Selected
162	-.46143	4.72262	.8578	Selected
163	.52943	4.61392	.8666	Selected
164	.52943	4.59683	.8679	Selected

	ZSco01	ZSco02	ZSco03	ZSco04	ZSco05
124	1.18938	-1.13035	-1.15322	-.07461	-1.17424
125	1.18938	.88028	-1.15322	-1.02683	-.15141
126	1.18938	.88028	.96607	1.35370	-.15141
127	-.83659	-1.13035	-.09358	-.31267	.61572
128	-.83659	-1.13035	-1.15322	-1.02683	-.79068
129	1.18938	.88028	1.49589	.87760	-1.21686
130	-.83659	-1.13035	1.76080	.87760	-1.28789
131	-.83659	.88028	.96607	.87760	-1.21686
132	-.83659	.88028	.17133	.63955	-.15141
133	-.83659	-1.13035	.70116	-.31267	-.15141
134	-.83659	.88028	.43625	-.55072	-1.42995
135	-.83659	-1.13035	1.49589	-.07461	-.15141
136	-.83659	.88028	.17133	-.31267	-.40712
137	1.18938	.88028	-.35849	.40149	.10430
138	1.18938	.88028	.43625	-1.26488	-.36450
139	1.18938	.88028	1.49589	.16344	-.79068
140	1.18938	.88028	-.35849	.63955	-.66283
141	-.83659	.88028	.70116	-.55072	1.34023
142	-.83659	-1.13035	-.09358	-.07461	-.15141
143	1.18938	-1.13035	-.35849	.40149	1.34023
144	-.83659	.88028	1.49589	.16344	.06168
145	1.18938	.88028	-.62340	-.78877	-.15141
146	-.83659	.88028	.17133	.63955	.48787
147	1.18938	.88028	-.09358	.16344	-.91853
148	-.83659	-1.13035	-1.15322	-1.02683	1.38285
149	-.83659	.88028	.17133	-.55072	-.15141
150	1.18938	-1.13035	-.09358	-.07461	-.15141
151	1.18938	.88028	-1.41813	-1.26488	.10430
152	1.18938	.88028	.17133	-.78877	-.47104
153	-.83659	.88028	-.62340	-.55072	-.15141
154	1.18938	-1.13035	-.35849	-.07461	-.15141
155	1.18938	.88028	-.88831	-.07461	-1.17424
156	-.83659	-1.13035	-.88831	-.55072	-.88200
157	-.83659	-1.13035	.17133	.16344	-1.42995
158	1.18938	.88028	.70116	-.31267	.16823
159	-.83659	-1.13035	-1.68304	-1.02683	.61572
160	-.83659	.88028	-.09358	-.31267	1.28696
161	-.83659	-1.13035	-.35849	-1.26488	2.40568
162	-.83659	-1.13035	-.35849	-.31267	-1.06465
163	-.83659	.88028	-1.41813	-.31267	1.34023
164	-.83659	-1.13035	-.62340	-.55072	-1.42995

	ZSco06	ZSco07	ZSco08	ZSco09	ZSco10
124	-1.64732	-.52734	-.93436	-1.32320	-.59710
125	-1.43083	-.88442	-.93436	-.17582	-.40750
126	-.34842	.24635	.39172	-.40529	1.48851
127	-.34842	-.88442	-.68572	-1.32320	-1.35550
128	-1.21435	1.13906	-.02268	.16840	-.78670
129	1.59993	.00829	1.05476	.85682	.73010
130	.73400	.42489	-.18844	.05366	.91971
131	-.88963	-1.36054	.55748	1.43051	.91971
132	-.88963	.48440	.47460	.74209	-.40750
133	.30103	.72246	.14308	.05366	-1.54510
134	-.34842	.18683	.30884	.97156	.73010
135	.30103	.06780	.88900	1.08630	.73010
136	.40927	.90100	-.60284	-.06108	.91971
137	-.02369	1.31760	.30884	.62735	.16130
138	-.45666	-.70588	-.35420	-.06108	-.40750
139	-.24018	.42489	-.18844	.16840	.16130
140	-1.10611	.24635	-.60284	-.86424	1.10931
141	.84224	-.22977	.14308	.62735	-1.35550
142	.30103	2.21032	-.10556	-.17582	-.21790
143	-.24018	-1.24151	.88900	.62735	-.97630
144	-.02369	.06780	1.05476	1.20104	1.10931
145	-.45666	-.17025	.47460	1.31578	1.10931
146	.95048	-.05122	1.46916	1.43051	1.67811
147	-.99787	.84149	1.22052	1.08630	.73010
148	.19279	1.43663	-.27132	-.74951	.73010
149	.30103	-.52734	1.38628	1.65999	1.29891
150	.62575	-.46782	.47460	-.40529	.35090
151	-.13194	.84149	.64036	.05366	.73010
152	.62575	.36538	.30884	-.40529	-.02830
153	-1.21435	.66295	-1.51452	-1.55267	-.59710
154	.08455	-.88442	.06020	.62735	-.21790
155	-.78138	.24635	1.22052	.97156	.54050
156	-1.10611	.30586	-.18844	-.63477	.35090
157	-.34842	-1.89617	.97188	.85682	.91971
158	-.78138	.12732	.88900	.85682	1.29891
159	-.78138	-.58685	-.60284	-1.20846	-.21790
160	-.13194	-1.18199	.72324	.28313	-.21790
161	-.78138	.24635	.47460	-.17582	-1.16590
162	-.99787	-.94394	.64036	-.29056	-1.16590
163	.62575	-.17025	.55748	.51261	-.02830
164	.84224	-.94394	1.13764	1.08630	.73010

	ZSco11	ZSco12	ZSco13	ZSco14	ZSco15
124	-.25524	-.32353	-.27530	-1.68541	-.21456
125	-.05484	-1.31634	.31674	.48232	.46318
126	1.74878	.17288	1.50080	-.60155	1.14093
127	-1.65805	-1.31634	-1.45936	.48232	-.89231
128	-1.05685	.17288	-.27530	.48232	-.89231
129	-.25524	1.16569	.31674	.48232	1.14093
130	.74677	-.81994	-.27530	-.60155	.46318
131	.74677	.17288	-.86733	.48232	-.89231
132	-1.05685	-.32353	.90877	.48232	-.21456
133	-1.25725	-.32353	.31674	.48232	.46318
134	1.14758	-.32353	-.27530	.48232	.46318
135	.54637	.17288	1.50080	.48232	.46318
136	.74677	.66929	1.50080	.48232	.46318
137	-.45564	-.32353	.90877	.48232	1.81867
138	-.25524	-.81994	.90877	.48232	.46318
139	-.05484	.66929	.90877	.48232	.46318
140	1.54838	-.32353	.31674	.48232	1.14093
141	-1.45765	.66929	1.50080	.48232	1.14093
142	-.45564	.17288	.31674	-.60155	-.89231
143	-1.25725	1.16569	-1.45936	.48232	1.14093
144	1.34798	.66929	.90877	-.60155	.46318
145	.74677	.66929	.90877	.48232	.46318
146	1.54838	1.16569	.90877	.48232	-.89231
147	.34597	1.16569	-.27530	.48232	.46318
148	.54637	.17288	.31674	.48232	.46318
149	1.34798	1.16569	.31674	.48232	-.89231
150	1.14758	-.32353	-.86733	.48232	-.89231
151	.54637	.66929	-1.45936	.48232	-.89231
152	.94717	.66929	-.86733	.48232	-.89231
153	.14557	-1.81275	-.86733	.48232	-.89231
154	-.25524	1.16569	-1.45936	.48232	-.89231
155	.34597	.66929	.31674	-.60155	.46318
156	.14557	.66929	.31674	.48232	-.89231
157	.74677	.66929	.90877	.48232	1.14093
158	1.34798	.66929	-.86733	.48232	-.89231
159	-.25524	-.32353	-.27530	.48232	-.89231
160	-1.25725	.17288	1.50080	.48232	.46318
161	-.85645	.17288	.	.	.
162	-.85645	.17288	-1.45936	.48232	-.89231
163	-.05484	.17288	.90877	-.60155	.46318
164	.74677	1.16569	-1.45936	.48232	-.89231

	ZSco16
124	-1.44075
125	.52614
126	.52614
127	-1.44075
128	.52614
129	-.45730
130	.52614
131	.52614
132	.52614
133	.52614
134	.52614
135	.52614
136	-.45730
137	.52614
138	-.45730
139	-.45730
140	-.45730
141	.52614
142	-.45730
143	.52614
144	-1.44075
145	-.45730
146	.52614
147	.52614
148	.52614
149	.52614
150	.52614
151	.52614
152	.52614
153	.52614
154	.52614
155	-.45730
156	.52614
157	.52614
158	.52614
159	.52614
160	.52614
161	.
162	-.45730
163	.52614
164	.52614

	gender	PARENT_CYCLE_TRAINING_...	BIKEABILITY_L2_CODE	Can_Cycle	Cycle_Access	POSITIVE_ATTITUDE_CHILD	POSITIVE_ATTITUDE_PARENT	NEGATIVE_EXPERIENCES
165	Male	1	0	Yes	Yes	21	16	1.60
166	Prefer n	0	0	Yes	No	19	15	1.00
167	Male	1	1	Yes	Yes	21	22	.50
168	Female	0	0	Yes	Yes	19	13	1.60
169	Male	1	1	Yes	Yes	22	21	1.80
170	Male	0	0	Yes	Yes	15	18	.
171	Female	0	1	Yes	Yes	19	21	.80
172	Male	0	1	Yes	Yes	19	20	.50
173	Female	0	0	Yes	Yes	17	13	.83
174	Female	1	1	Yes	Yes	17	14	2.60
175	Male	0	1	Yes	Yes	21	17	1.25
176	Male	0	1	Yes	Yes	20	19	.60
177	Male	1	1	Yes	No	20	17	1.00
178	Female	0	0	Yes	Yes	18	18	1.00
179	Female	0	1	Yes	No	14	14	.50
180	Female	0	1	Yes	No	17	17	1.00
181	Male	0	0	Yes	Yes	17	14	2.00
182	Male	1	1	Yes	Yes	20	21	1.20
183	Male	0	0	Yes	No	20	21	.50
184	Female	0	1	Yes	Yes	18	21	1.17
185	Female	1	1	Yes	Yes	18	21	.44
186	Male	0	1	Yes	Yes	18	15	2.40
187	Female	1	0	Yes	Yes	20	20	1.00
188	Female	1	1	Yes	Yes	17	20	1.00
189	Male	0	0	Yes	Yes	15	12	1.25
190	Female	1	1	Yes	Yes	19	21	1.67
191	Male	1	1	Yes	Yes	17	13	.67
192	Female	0	1	Yes	Yes	22	19	1.29
193	Male	1	1	Yes	Yes	16	16	1.67
194	Female	0	0	Yes	No	20	16	.00
195	Male	0	1	Yes	Yes	19	18	.80
196	Male	0	1	Yes	Yes	15	15	2.00
197	Male	0	1	Yes	Yes	21	18	.67
198	Female	1	0	Yes	Yes	16	16	2.33
199	Female	1	1	Yes	Yes	20	18	.67
200	Male	1	1	Yes	Yes	18	16	.70
201	Male	0	0	Yes	Yes	17	18	1.14

SPSS DATA - 240325.sav

	MVPA	SEDENTARY	LIFE_SATISFACTION	WELLBEING	SATISFACTION_NOW	SATISFACTION_TEN_YEARS	Life_Satisfaction_Single_Item	Child_Cycling_Frequency
165	8	12	47	62	29	28	9.00	6.00
166	8	40	47	57	34	35	9.00	2.00
167	19	20	60	67	31	31	10.00	5.00
168	19	42	44	58	28	28	7.00	4.00
169	19	38	54	56	32	32	9.00	6.00
170	19	38	21	48	26	26	5.00	4.00
171	14	52	38	56	35	36	8.00	3.00
172	16	22	56	58	29	28	9.00	5.00
173	9	22	50	60	28	29	8.00	1.00
174	11	35	52	57	32	32	9.00	4.00
175	16	56	43	55	30	29	9.00	4.00
176	19	43	48	67	32	33	10.00	6.00
177	13	15	41	54	30	27	8.00	5.00
178	11	48	29	46	23	28	5.00	3.00
179	17	28	36	49	20	25	7.00	1.00
180	17	50	47	60	24	24	8.00	3.00
181	16	24	52	64	25	25	9.00	3.00
182	7	38	44	55	31	30	9.00	6.00
183	22	52	41	51	28	29	7.00	1.00
184	10	43	37	56	33	33	7.00	3.00
185	14	32	49	61	24	25	9.00	5.00
186	24	40	27	51	26	26	7.00	3.00
187	20	36	48	65	27	25	10.00	4.00
188	16	31	51	59	25	26	9.00	6.00
189	17	28	43	54	27	28	9.00	4.00
190	21	20	47	60	29	29	9.00	3.00
191	20	27	31	53	27	28	7.00	5.00
192	25	31	39	60	27	26	8.00	5.00
193	13	32	52	59	29	30	9.00	4.00
194	19	45	49	61	28	27	9.00	1.00
195	21	40	45	53	32	33	8.00	4.00
196	10	40	31	49	30	31	6.00	3.00
197	23	34	48	64	26	26	9.00	5.00
198	20	34	40	60	25	25	8.00	3.00
199	17	49	47	60	32	32	9.00	4.00
200	17	37	42	54	26	25	7.00	6.00
201	18	30	40	52	27	27	8.00	4.00

	Child_Walking_Frequency	Parent_Cycling_Frequency	Parent_Walking_Frequency	ZPARENT_CYCLE_TRAINING_CODE
165	6.00	1.00	6.00	1.20943
166	6.00	1.00	6.00	-.82281
167	6.00	4.00	6.00	1.20943
168	6.00	1.00	5.00	-.82281
169	5.00	4.00	6.00	1.20943
170	6.00	3.00	6.00	-.82281
171	6.00	3.00	6.00	-.82281
172	5.00	4.00	5.00	-.82281
173	6.00	1.00	6.00	-.82281
174	6.00	3.00	6.00	1.20943
175	5.00	2.00	6.00	-.82281
176	5.00	1.00	3.00	-.82281
177	6.00	1.00	6.00	1.20943
178	6.00	3.00	6.00	-.82281
179	6.00	1.00	6.00	-.82281
180	6.00	5.00	6.00	-.82281
181	6.00	2.00	6.00	-.82281
182	5.00	2.00	4.00	1.20943
183	5.00	1.00	5.00	-.82281
184	6.00	3.00	6.00	-.82281
185	5.00	3.00	5.00	1.20943
186	6.00	3.00	5.00	-.82281
187	6.00	4.00	6.00	1.20943
188	5.00	4.00	6.00	1.20943
189	6.00	1.00	6.00	-.82281
190	6.00	3.00	6.00	1.20943
191	5.00	4.00	6.00	1.20943
192	6.00	1.00	5.00	-.82281
193	5.00	3.00	6.00	1.20943
194	6.00	1.00	6.00	-.82281
195	6.00	3.00	6.00	-.82281
196	6.00	2.00	6.00	-.82281
197	6.00	5.00	6.00	-.82281
198	6.00	1.00	6.00	1.20943
199	6.00	3.00	6.00	1.20943
200	6.00	4.00	6.00	1.20943
201	6.00	4.00	6.00	-.82281

	ZBIKEABILITY_L2_CODE	ZPOSITIVE_ATTITUDE_CHILD	ZPOSITIVE_ATTITUDE_PARENT
165	-1.12763	.70599	-.31978
166	-1.12763	.17194	-.55904
167	.88249	.70599	1.11574
168	-1.12763	.17194	-1.03755
169	.88249	.97302	.87649
170	-1.12763	-.89617	.15872
171	.88249	.17194	.87649
172	.88249	.17194	.63723
173	-1.12763	-.36212	-1.03755
174	.88249	-.36212	-.79829
175	.88249	.70599	-.08053
176	.88249	.43897	.39798
177	.88249	.43897	-.08053
178	-1.12763	-.09509	.15872
179	.88249	-1.16320	-.79829
180	.88249	-.36212	-.08053
181	-1.12763	-.36212	-.79829
182	.88249	.43897	.87649
183	-1.12763	.43897	.87649
184	.88249	-.09509	.87649
185	.88249	-.09509	.87649
186	.88249	-.09509	-.55904
187	-1.12763	.43897	.63723
188	.88249	-.36212	.63723
189	-1.12763	-.89617	-1.27680
190	.88249	.17194	.87649
191	.88249	-.36212	-1.03755
192	.88249	.97302	.39798
193	.88249	-.62914	-.31978
194	-1.12763	.43897	-.31978
195	.88249	.17194	.15872
196	.88249	-.89617	-.55904
197	.88249	.70599	.15872
198	-1.12763	-.62914	-.31978
199	.88249	.43897	.15872
200	.88249	-.09509	-.31978
201	-1.12763	-.36212	.15872

	ZNEGATIVE_EXPERIENCES	ZMVPA	ZSEDENTARY	ZLIFE_SATISFACTION
165	.60827	-.89486	-1.40978	.65282
166	-.16159	-.89486	.25595	.65282
167	-.80315	.30271	-.93386	1.71690
168	.60827	.30271	.37494	.40726
169	.86489	.30271	.13697	1.22578
170	.	.30271	.13697	-1.47533
171	-.41821	-.24164	.96984	-.08385
172	-.80315	-.02390	-.81488	1.38949
173	-.37544	-.78599	-.81488	.89838
174	1.89137	-.56825	-.04150	1.06208
175	.15918	-.02390	1.20780	.32541
176	-.67483	.30271	.43443	.73467
177	-.16159	-.35051	-1.23131	.16171
178	-.16159	-.56825	.73188	-.82052
179	-.80315	.08497	-.45793	-.24755
180	-.16159	.08497	.85086	.65282
181	1.12151	-.02390	-.69589	1.06208
182	.09503	-1.00373	.13697	.40726
183	-.80315	.62932	.96984	.16171
184	.05226	-.67712	.43443	-.16570
185	-.87443	-.24164	-.21997	.81652
186	1.63475	.84707	.25595	-.98422
187	-.16159	.41158	.01799	.73467
188	-.16159	-.02390	-.27946	.98023
189	.15918	.08497	-.45793	.32541
190	.69381	.52045	-.93386	.65282
191	-.58929	.41158	-.51742	-.65681
192	.20501	.95594	-.27946	-.00200
193	.69381	-.35051	-.21997	1.06208
194	-1.44470	.30271	.55341	.81652
195	-.41821	.52045	.25595	.48912
196	1.12151	-.67712	.25595	-.65681
197	-.58929	.73820	-.10099	.73467
198	1.54921	.41158	-.10099	.07986
199	-.58929	.08497	.79137	.65282
200	-.54652	.08497	.07748	.24356
201	.02171	.19384	-.33895	.07986

	ZWELLBEING	ZSATISFACTION_NOW	ZSATISFACTION_TEN_YEARS
165	.84796	.35074	-.04312
166	.27350	1.28694	1.30211
167	1.42242	.72522	.53341
168	.38839	.16349	-.04312
169	.15861	.91246	.72558
170	-.76053	-.21099	-.42748
171	.15861	1.47418	1.49429
172	.38839	.35074	-.04312
173	.61818	.16349	.14905
174	.27350	.91246	.72558
175	.04372	.53798	.14905
176	1.42242	.91246	.91776
177	-.07118	.53798	-.23530
178	-.99031	-.77271	-.04312
179	-.64564	-1.33444	-.61965
180	.61818	-.58547	-.81183
181	1.07774	-.39823	-.61965
182	.04372	.72522	.34123
183	-.41585	.16349	.14905
184	.15861	1.09970	.91776
185	.73307	-.58547	-.61965
186	-.41585	-.21099	-.42748
187	1.19264	-.02375	-.61965
188	.50328	-.39823	-.42748
189	-.07118	-.02375	-.04312
190	.61818	.35074	.14905
191	-.18607	-.02375	-.04312
192	.61818	-.02375	-.42748
193	.50328	.35074	.34123
194	.73307	.16349	-.23530
195	-.18607	.91246	.91776
196	-.64564	.53798	.53341
197	1.07774	-.21099	-.42748
198	.61818	-.39823	-.61965
199	.61818	.91246	.72558
200	-.07118	-.21099	-.61965
201	-.30096	-.02375	-.23530

	ZLife_Satisfaction_Single_Item	ZChild_Cycling_Frequency
165	.66396	1.50175
166	.66396	-.85939
167	1.15357	.91147
168	-.31526	.32118
169	.66396	1.50175
170	-1.29448	.32118
171	.17435	-.26910
172	.66396	.91147
173	.17435	-1.44967
174	.66396	.32118
175	.66396	.32118
176	1.15357	1.50175
177	.17435	.91147
178	-1.29448	-.26910
179	-.31526	-1.44967
180	.17435	-.26910
181	.66396	-.26910
182	.66396	1.50175
183	-.31526	-1.44967
184	-.31526	-.26910
185	.66396	.91147
186	-.31526	-.26910
187	1.15357	.32118
188	.66396	1.50175
189	.66396	.32118
190	.66396	-.26910
191	-.31526	.91147
192	.17435	.91147
193	.66396	.32118
194	.66396	-1.44967
195	.17435	.32118
196	-.80487	-.26910
197	.66396	.91147
198	.17435	-.26910
199	.66396	.32118
200	-.31526	1.50175
201	.17435	.32118

	ZChild_Walking_Frequency	ZParent_Cycling_Frequency
165	.48168	-.88951
166	.48168	-.88951
167	.48168	1.15468
168	.48168	-.88951
169	-.61013	1.15468
170	.48168	.47328
171	.48168	.47328
172	-.61013	1.15468
173	.48168	-.88951
174	.48168	.47328
175	-.61013	-.20811
176	-.61013	-.88951
177	.48168	-.88951
178	.48168	.47328
179	.48168	-.88951
180	.48168	1.83607
181	.48168	-.20811
182	-.61013	-.20811
183	-.61013	-.88951
184	.48168	.47328
185	-.61013	.47328
186	.48168	.47328
187	.48168	1.15468
188	-.61013	1.15468
189	.48168	-.88951
190	.48168	.47328
191	-.61013	1.15468
192	.48168	-.88951
193	-.61013	.47328
194	.48168	-.88951
195	.48168	.47328
196	.48168	-.20811
197	.48168	1.83607
198	.48168	-.88951
199	.48168	.47328
200	.48168	1.15468
201	.48168	1.15468

	ZParent_Walking_Frequency	MAH_2	Probability_MAH_2	filter_$
165	.52943	4.58767	.8687	Selected
166	.52943	4.43646	.8804	Selected
167	.52943	4.41434	.8821	Selected
168	-.46143	4.40548	.8828	Selected
169	.52943	4.38231	.8845	Selected
170	.52943	4.29161	.8912	Selected
171	.52943	4.27152	.8927	Selected
172	-.46143	4.22716	.8958	Selected
173	.52943	4.21247	.8969	Selected
174	.52943	3.73633	.9279	Selected
175	.52943	3.71123	.9294	Selected
176	-2.44316	3.69476	.9303	Selected
177	.52943	3.63889	.9335	Selected
178	.52943	3.63258	.9339	Selected
179	.52943	3.55540	.9382	Selected
180	.52943	3.53666	.9392	Selected
181	.52943	3.36641	.9480	Selected
182	-1.45230	3.35846	.9484	Selected
183	-.46143	3.28191	.9521	Selected
184	.52943	3.25571	.9533	Selected
185	-.46143	3.18208	.9566	Selected
186	-.46143	3.13292	.9588	Selected
187	.52943	3.09113	.9606	Selected
188	.52943	2.99015	.9647	Selected
189	.52943	2.88591	.9686	Selected
190	.52943	2.59947	.9781	Selected
191	.52943	2.57561	.9788	Selected
192	-.46143	2.51894	.9804	Selected
193	.52943	2.31272	.9855	Selected
194	.52943	2.26919	.9865	Selected
195	.52943	2.24118	.9871	Selected
196	.52943	2.15974	.9887	Selected
197	.52943	2.03857	.9908	Selected
198	.52943	2.03395	.9909	Selected
199	.52943	1.97713	.9918	Selected
200	.52943	1.71322	.9952	Selected
201	.52943	1.17994	.9989	Selected

	ZSco01	ZSco02	ZSco03	ZSco04	ZSco05
165	1.18938	-1.13035	.70116	-.31267	.61572
166	-.83659	-1.13035	.17133	-.55072	-.15141
167	1.18938	.88028	.70116	1.11565	-.79068
168	-.83659	-1.13035	.17133	-1.02683	.61572
169	1.18938	.88028	.96607	.87760	.87143
170	-.83659	-1.13035	-.88831	.16344	.
171	-.83659	.88028	.17133	.87760	-.40712
172	-.83659	.88028	.17133	.63955	-.79068
173	-.83659	-1.13035	-.35849	-1.02683	-.36450
174	1.18938	.88028	-.35849	-.78877	1.89427
175	-.83659	.88028	.70116	-.07461	.16823
176	-.83659	.88028	.43625	.40149	-.66283
177	1.18938	.88028	.43625	-.07461	-.15141
178	-.83659	-1.13035	-.09358	.16344	-.15141
179	-.83659	.88028	-1.15322	-.78877	-.79068
180	-.83659	.88028	-.35849	-.07461	-.15141
181	-.83659	-1.13035	-.35849	-.78877	1.12714
182	1.18938	.88028	.43625	.87760	.10430
183	-.83659	-1.13035	.43625	.87760	-.79068
184	-.83659	.88028	-.09358	.87760	.06168
185	1.18938	.88028	-.09358	.87760	-.86171
186	-.83659	.88028	-.09358	-.55072	1.63856
187	1.18938	-1.13035	.43625	.63955	-.15141
188	1.18938	.88028	-.35849	.63955	-.15141
189	-.83659	-1.13035	-.88831	-1.26488	.16823
190	1.18938	.88028	.17133	.87760	.70096
191	1.18938	.88028	-.35849	-1.02683	-.57759
192	-.83659	.88028	.96607	.40149	.21389
193	1.18938	.88028	-.62340	-.31267	.70096
194	-.83659	-1.13035	.43625	-.31267	-1.42995
195	-.83659	.88028	.17133	.16344	-.40712
196	-.83659	.88028	-.88831	-.55072	1.12714
197	-.83659	.88028	.70116	.16344	-.57759
198	1.18938	-1.13035	-.62340	-.31267	1.55332
199	1.18938	.88028	.43625	.16344	-.57759
200	1.18938	.88028	-.09358	-.31267	-.53497
201	-.83659	-1.13035	-.35849	.16344	.03124

	ZSco06	ZSco07	ZSco08	ZSco09	ZSco10
165	-.88963	-1.42005	.64036	.85682	.35090
166	-.88963	.24635	.64036	.28313	1.29891
167	.30103	-.94394	1.71780	1.43051	.73010
168	.30103	.36538	.39172	.39787	.16130
169	.30103	.12732	1.22052	.16840	.91971
170	.30103	.12732	-1.51452	-.74951	-.21790
171	-.24018	.96052	-.10556	.16840	1.48851
172	-.02369	-.82491	1.38628	.39787	.35090
173	-.78138	-.82491	.88900	.62735	.16130
174	-.56490	-.05122	1.05476	.28313	.91971
175	-.02369	1.19858	.30884	.05366	.54050
176	.30103	.42489	.72324	1.43051	.91971
177	-.34842	-1.24151	.14308	-.06108	.54050
178	-.56490	.72246	-.85148	-.97898	-.78670
179	.08455	-.46782	-.27132	-.63477	-1.35550
180	.08455	.84149	.64036	.62735	-.59710
181	-.02369	-.70588	1.05476	1.08630	-.40750
182	-.99787	.12732	.39172	.05366	.73010
183	.62575	.96052	.14308	-.40529	.16130
184	-.67314	.42489	-.18844	.16840	1.10931
185	-.24018	-.22977	.80612	.74209	-.59710
186	.84224	.24635	-1.01724	-.40529	-.21790
187	.40927	.00829	.72324	1.20104	-.02830
188	-.02369	-.28928	.97188	.51261	-.40750
189	.08455	-.46782	.30884	-.06108	-.02830
190	.51751	-.94394	.64036	.62735	.35090
191	.40927	-.52734	-.68572	-.17582	-.02830
192	.95048	-.28928	-.02268	.62735	-.02830
193	-.34842	-.22977	1.05476	.51261	.35090
194	.30103	.54392	.80612	.74209	.16130
195	.51751	.24635	.47460	-.17582	.91971
196	-.67314	.24635	-.68572	-.63477	.54050
197	.73400	-.11074	.72324	1.08630	-.21790
198	.40927	-.11074	.06020	.62735	-.40750
199	.08455	.78198	.64036	.62735	.91971
200	.08455	.06780	.22596	-.06108	-.21790
201	.19279	-.34880	.06020	-.29056	-.02830

	ZSco11	ZSco12	ZSco13	ZSco14	ZSco15
165	-.05484	.66929	1.50080	.48232	-.89231
166	1.34798	.66929	-.86733	.48232	-.89231
167	.54637	1.16569	.90877	.48232	1.14093
168	-.05484	-.32353	.31674	.48232	-.89231
169	.74677	.66929	1.50080	-.60155	1.14093
170	-.45564	-1.31634	.31674	.48232	.46318
171	1.54838	.17288	-.27530	.48232	.46318
172	-.05484	.66929	.90877	-.60155	1.14093
173	.14557	.17288	-1.45936	.48232	-.89231
174	.74677	.66929	.31674	.48232	.46318
175	.14557	.66929	.31674	-.60155	-.21456
176	.94717	1.16569	1.50080	-.60155	-.89231
177	-.25524	.17288	.90877	.48232	-.89231
178	-.05484	-1.31634	-.27530	.48232	.46318
179	-.65604	-.32353	-1.45936	.48232	-.89231
180	-.85645	.17288	-.27530	.48232	1.81867
181	-.65604	.66929	-.27530	.48232	-.21456
182	.34597	.66929	1.50080	-.60155	-.21456
183	.14557	-.32353	-1.45936	-.60155	-.89231
184	.94717	-.32353	-.27530	.48232	.46318
185	-.65604	.66929	.90877	-.60155	.46318
186	-.45564	-.32353	-.27530	.48232	.46318
187	-.65604	1.16569	.31674	.48232	1.14093
188	-.45564	.66929	1.50080	-.60155	1.14093
189	-.05484	.66929	.31674	.48232	-.89231
190	.14557	.66929	-.27530	.48232	.46318
191	-.05484	-.32353	.90877	-.60155	1.14093
192	-.45564	.17288	.90877	.48232	-.89231
193	.34597	.66929	.31674	-.60155	.46318
194	-.25524	.66929	-1.45936	.48232	-.89231
195	.94717	.17288	.31674	.48232	.46318
196	.54637	-.81994	-.27530	.48232	-.21456
197	-.45564	.66929	.90877	.48232	1.81867
198	-.65604	.17288	-.27530	.48232	-.89231
199	.74677	.66929	.31674	.48232	.46318
200	-.65604	-.32353	1.50080	.48232	1.14093
201	-.25524	.17288	.31674	.48232	1.14093

	ZSco16
165	.52614
166	.52614
167	.52614
168	-.45730
169	.52614
170	.52614
171	.52614
172	-.45730
173	.52614
174	.52614
175	.52614
176	-2.42420
177	.52614
178	.52614
179	.52614
180	.52614
181	.52614
182	-1.44075
183	-.45730
184	.52614
185	-.45730
186	-.45730
187	.52614
188	.52614
189	.52614
190	.52614
191	.52614
192	-.45730
193	.52614
194	.52614
195	.52614
196	.52614
197	.52614
198	.52614
199	.52614
200	.52614
201	.52614
